# Removal of Hepatitis C Virus-Infected Cells by a Zymogenized Bacterial Toxin

**DOI:** 10.1371/journal.pone.0032320

**Published:** 2012-02-16

**Authors:** Assaf Shapira, Shiran Shapira, Meital Gal-Tanamy, Romy Zemel, Ran Tur-Kaspa, Itai Benhar

**Affiliations:** 1 Department of Molecular Microbiology and Biotechnology, The George S. Wise Faculty of Life Sciences, Tel-Aviv University, Ramat Aviv, Israel; 2 Molecular Hepatology Research Laboratory, Sackler School of Medicine, Felsenstein Medical Research Center, Tel-Aviv University, Petah Tikva, Israel; 3 The Integrated Cancer Prevention Center, Tel Aviv Medical Center, Tel-Aviv, Israel; 4 Sackler School of Medicine, Tel-Aviv University, Ramat Aviv, Israel; 5 Department of Medicine D and Liver Institute, Rabin Medical Center, Beilinson Campus, Petah Tikva, Israel; Pohang University of Science and Technology, Korea

## Abstract

Hepatitis C virus (HCV) infection is a major cause of chronic liver disease and has become a global health threat. No HCV vaccine is currently available and treatment with antiviral therapy is associated with adverse side effects. Moreover, there is no preventive therapy for recurrent hepatitis C post liver transplantation. The NS3 serine protease is necessary for HCV replication and represents a prime target for developing anti HCV therapies. Recently we described a therapeutic approach for eradication of HCV infected cells that is based on protein delivery of two NS3 protease-activatable recombinant toxins we named “zymoxins”. These toxins were inactivated by fusion to rationally designed inhibitory peptides via NS3-cleavable linkers. Once delivered to cells where NS3 protease is present, the inhibitory peptide is removed resulting in re-activation of cytotoxic activity. The zymoxins we described suffered from two limitations: they required high levels of protease for activation and had basal activities in the un-activated form that resulted in a narrow potential therapeutic window. Here, we present a solution that overcame the major limitations of the “first generation zymoxins” by converting MazF ribonuclease, the toxic component of the *E. coli* chromosomal MazEF toxin-antitoxin system, into an NS3-activated zymoxin that is introduced to cells by means of gene delivery. We constructed an expression cassette that encodes for a single polypeptide that incorporates both the toxin and a fragment of its potent natural antidote, MazE, linked via an NS3-cleavable linker. While covalently paired to its inhibitor, the ribonuclease is well tolerated when expressed in naïve, healthy cells. In contrast, activating proteolysis that is induced by even low levels of NS3, results in an eradication of NS3 expressing model cells and HCV infected cells. Zymoxins may thus become a valuable tool in eradicating cells infected by intracellular pathogens that express intracellular proteases.

## Introduction

Hepatitis C virus (HCV) is a small, enveloped RNA virus belonging to the *Hepacivirus* genus of the *Flaviviridae* family. HCV has been recognized as a major cause of chronic liver disease and affects approximately 200 million people worldwide at the present time. Persistent infection is associated with the development of chronic hepatitis, cirrhosis and hepatocellular carcinoma. A protective vaccine for HCV is not yet available and even the most recent combination of antiviral therapy is often poorly tolerated [1]. The HCV genome encodes one large open reading frame that is translated as a polyprotein which is proteolytically processed to yield the viral structural and nonstructural (NS) proteins. The non-structural proteins include the p7 ion channel, the NS2-3 protease, the NS3 serine protease/RNA helicase and its co-factor NS4A, the NS4B and NS5A proteins and the NS5B RNA-dependent RNA polymerase (RdRp) [2], [3]. Two virally encoded proteases participate in polyprotein processing, the NS2-3 autoprotease (which cleaves in *cis* at the NS2-3 junction) and the NS3-4A serine protease (which cleaves at four downstream NS protein junctions). NS3 is an extensively studied HCV protein that possesses multiple enzymatic activities that are essential for HCV replication, making NS3-4A the most attractive target for anti HCV drug development [4]. The N-terminus of NS3, in complex with its endoplasmic reticulum (ER) membrane-anchored co-factor NS4A, primarily functions as a serine protease, which cleaves the viral polyprotein precursor downstream to NS3. The remaining 2/3 of the protein has a helicase and NTPase activities, both of which are essential for HCV replication [5].

Zymogens are inactive enzyme precursors that are converted to their active form following a biochemical modification, such as proteolytic processing. Among the known and important groups of enzymes that are proteolytically activated are secreted digestive enzymes like pepsin and trypsin [6], [7], the cysteine aspartic acid proteases (caspases) which play an essential role at various stages of the apoptotic process [8]; and blood coagulating factors [9].

Recently we described a proof of concept for potential new anti-viral agents that can specifically eradicate virally infected cells (thus limiting virus production and spread). These agents, which we named “zymoxins” (for “zymogenized toxins”), were composed of a fusion between the binding and translocation domains of *Pseudomonas* exotoxin A and NS3-activable modified catalytic domains of the bacterial or the plant toxins diphtheria toxin (DTA) or ricin toxin (RTA), respectively. Upon binding and translocation into cells cytoplasm by virtue of the corresponding *Pseudomonas* exotoxin A domains, activation of these toxins is mediated by HCV-NS3 protease cleavage that separates between the toxic domains and a fused, rationally designed inhibitory peptide. These zymoxins showed a higher level of cytotoxicity when applied to NS3 expressing cells or to HCV infected cells, demonstrating a potential therapeutic window. Still, the zymoxins we described had two limitations: first, they required high levels of NS3 protease for efficient activation, and second, they were not totally inactive in the zymogenized state, and their basal cytotoxic activity without proteolytic activation resulted in quite narrow therapeutic windows [10]. Apparently, the zymoxins were only partially inhibited by the rationally designed inhibitory peptides.

Toxic proteins that are completely inhibited by a natural inhibitor can be found in bacterial “toxin-antitoxin” systems. Natural bacterial plasmids ensure their survival within the bacterial host by replicating inside their host cell and using mechanisms that function to segregate them prior to cell division [11]. In addition, several low copy number plasmids use a unique system called “addiction module” or “toxin-antitoxin (TA) system” which functions to prevent the proliferation of plasmid-free cells by executing the so called “post-segregational killing effect”. The system consists of a pair of genes encoding for a stable toxin and an unstable antitoxin organized in a bicistronic operon that is transcriptionally autoregulated either by the toxin-antitoxin complex itself or by the antitoxin alone. When coexpressed in plasmid harboring cells, the antitoxin component interferes with the lethal action of the toxin. If a cell loses the plasmid, the cellular concentration of the labile antitoxin (that is degraded faster than the more stable toxin) is rapidly diminished, enabling the toxin to exert its action which eventually results in cell death (reviewed by [12], [13], [14], [15], [16]). Toxin-antitoxin systems have also been found integrated to the chromosome of various bacteria species where their function has been the subject of considerable speculation [14], [17], [18], [19], [20], [21]. One of the most studied “genomic” TA modules was found on the chromosome of *E. coli* as a negatively autoregulated bicistronic operon. The system, which is activated by several stress conditions, was denoted *MazEF* after its two active protein components: the long lived MazF toxin and the labile MazE antitoxin. MazF induced toxicity is executed by blocking *de-novo* protein synthesis through its endoribonuclease activity that catalyze the cleavage of single-stranded mRNAs at ACA sequences [22]. When coexpressed with MazF, the MazE antitoxin complexed with the toxin and a catalytically inactive heterohexamer is formed in which a MazE dimer is sandwiched between two MazF dimers (MazF_2_-MazE_2_-MazF_2_) (reviewed by [21], [23]). The crystal structure of the MazE-MazF complex indicates that the interactions between the toxin and the antitoxin are primarily mediated by the acidic C terminus of MazE which wraps around the MazF homodimer crossing the edge of the dimer interface [24]. Later on, Li *et al* had discovered that a short acidic peptide corresponding to MazE C-terminal 23 amino-acids, which they denoted “MazEp”, binds strongly to the homodimer of MazF which possesses two identical active sites. Interestingly, it was found that one inhibitory peptide, occupying a single active site on the MazF homodimer, affects the conformation of both sites that consequently become catalytically inactive. This unique mechanism also explains the inhibitory activity of MazE toward MazF at a 1∶2 molar ratio [25]. The discovery of the MazF mechanism of action was soon followed by demonstrations that it is also toxic to eukaryotic cells, causing Bak-dependent programmed cell death in mammalian cells, suggesting it may be used as a tool for gene therapy against diseases such as cancer and AIDS [26], [27].

Here we describe the design of a different zymoxin than those described in [10], based on NS3-activable MazF ribonuclease that is delivered as a transgene by an adenoviral vector. The delivered transgene encodes for a fusion between MazF, and a potent inhibitory peptide derived from its natural antidote MazE, through an NS3 cleavable linker. We show that the self-inhibited, zymogenized toxin is well tolerated when expressed in naïve cells. In contrast, in NS3 expressing or in HCV infected cells, NS3-mediated cleavage separates between the toxin and its inhibitor which results in inhibition of protein synthesis followed by death of the cells. Finally, we demonstrate that treatment with the MazF based zymoxin has a “curing effect” when applied to mixed culture of healthy and HCV infected cells, leading to specific eradication of the infected cell population.

## Results

### The construction of MazF-based zymoxin

**Figure 1:**
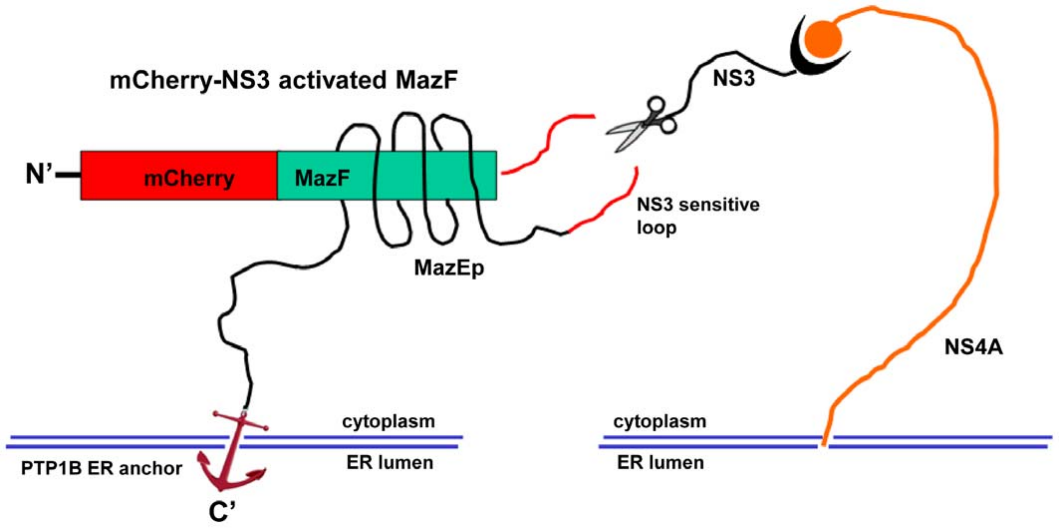
Schematic representation of the construct “mCherry-NS3 activated MazF” and hypothetical mechanism of activation by NS3 protease. The NS3-activated MazF zymoxin was constructed by fusing 5 elements in the following order (from the N terminus): monomeric red fluorescence protein mCherry, *E. coli* MazF ribonuclease, HCV P10-P10' NS3 cleavage sequence derived from 2a genotype (strain JFH1) NS5A/B junction, a short inhibitory peptide corresponding to MazE C-terminal 35 amino-acids (which encompass the 23 amino-acids inhibitory peptide (MazEp) that has been described by Li *et al.* [25]) and the C-terminal ER membrane anchor of the tyrosine phosphatase PTP1B [28]. After being anchored to the ER membrane, the NS3 cleavage site that is located between the ribonuclease and the inhibitory peptide in the “mCherry-NS3 activated MazF” construct (which is active as a dimer but for convenience is illustrated here in its monomeric form) is cleaved by the HCV- NS3 protease which is also localized to the cytoplasmic side of the ER membrane. The toxic ribonuclease, no longer covalently tethered to its ER-anchored inhibitor, is now free to diffuse to the cytoplasm (which lacks the antidote) and exert its destructive activity.

For the construction of NS3-activated MazF based zymoxin, the MazF coding sequence was fused through its C terminus to the HCV P10-P10' NS3 cleavage sequence derived from the 2a genotype (strain JFH1) NS5A/B junction. A short inhibitory peptide corresponding to MazE C-terminal 35 amino-acids (which encompass the 23 amino-acids inhibitory peptide (MazEp) that has been described by Li *et al.* [25]) was fused, preceded by a short flexible linker, to the C terminus of the MazF-NS3 cleavage site sequence. A flexible linker, followed by the C-terminal ER membrane anchor of the tyrosine phosphatase PTP1B [28], was than fused to the C terminus of the inhibitory peptide and the whole construct was fused through its N terminus to the monomeric red fluorescence protein mCherry [29] (**see** **Figure 1**). The rationale behind the design of this construct, which was denoted “mCherry-NS3 activated MazF”, was that the coupling between the ribonuclease and its antidote may enable high level of expression of the non-toxic fusion on the ER membrane of uninfected mammalian cells without causing any deleterious effect. In contrast, in HCV infected cells, the fusion protein is expected to colocalize with the ER membrane-bound viral NS3 protease (in infected cells, NS3 is localized to the cytosolic side of the ER membrane and membranes of ER-like modified compartments [30], [31], [32], [33], [34]). As a result, the NS3-cleavable linker between the toxin and its inhibitory peptide is expected to be cleaved. The toxic ribonuclease, no longer covalently tethered to its ER membrane-anchored inhibitor, is now free to diffuse into the cytoplasm (which lacks the antidote) and exert its cytotoxic activity. Finally, fusion to the fluorescent protein mCherry makes the whole construct trackable and facilitates the determination of its expression level and intracellular localization by fluorescence microscopy. As a control, an uncleavable construct (denoted “mCherry-uncleavable MazF”) was constructed in which the NS3 cleavage sequence was replaced by a mutated 14 amino acids cleavage sequence (P10-P4') from HCV genotype 1a NS5A/B junction in which the P3 valine was substituted by alanine, the P2 cysteine by glycine, the P1 cysteine by glycine and the P4' tyrosine by alanine. A schematic representation of the NS3-activated MazF-based zymoxin (“mCherry-NS3 activated MazF”) and the hypothetical mechanism of its cleavage by NS3 protease on the cytoplasmic side of the ER membrane are shown in **Figure 1**. The amino-acid sequence of the MazF based zymoxins can be found in **Text S1**.

### Expression of NS3 activated MazF is well tolerated by naïve cells

**Figure 2:**
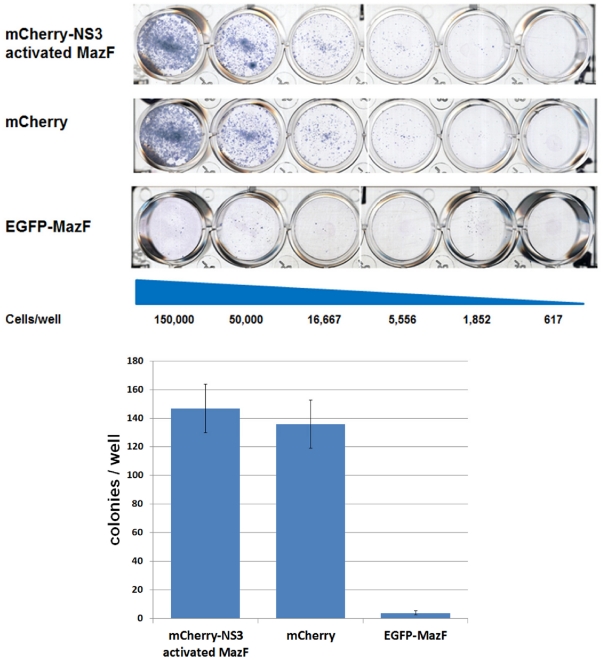
Colony formation assay for the assessment of “mCherry-NS3 activated MazF” cytotoxicity toward naïve cells. A day before transfection, 7.5×10^5^ HEK293 T-REx cells where seeded per well in 6 wells plate and subsequently transfected with 2 µg of plasmids encoding either mCherry-NS3 activated MazF, mCherry (only the fluorescent protein) or EGFP- MazF (where MazF is not fused to its inhibitory peptide). 48 hours later, transfection efficiency was assessed by fluorescence microscopy and was determined as equal between the plasmids. Transfected cells were than trypsinized, counted and seeded in 3 fold dilutions (starting from 150,000 cells/well) in 6 well plates and were incubated for 10 days in the presence of 1 mg/ml of G418 (to which all three plasmids confer resistance). Surviving colonies were fixed and stained with Giemsa (upper panel). Number of surviving Colonies from wells that were seeded with 5556 cells was determined by manual counting. Each bar represents the mean ± standard deviation (SD) of a set of data from two wells (lower panel).

To verify that expression of “mCherry-NS3 activated MazF” is tolerated by cells that do not express the NS3 protease, a colony formation assay was carried out. In this assay, the toxicity of an expressed transgene can be comparatively and qualitatively assessed by testing its effect on the competency of transfected cells to evolve into colonies under selection. HEK293 T-REx cells where transfected with plasmids encoding either mCherry-NS3 activated MazF, mCherry (only the fluorescent protein) or EGFP- MazF (where MazF is not fused to its inhibitory peptide). 48 hours (h) later, expression of the encoded transgenes was confirmed by fluorescence microscopy (data not shown) and transfected cells were seeded in 3 fold dilutions and were treated with G418 (to which all three plasmids confer resistance). After 10 days of selection, surviving colonies were stained. As shown in **Figure 2**, similar numbers of surviving colonies were observed when the cells were transfected with the plasmids encoding mCherry-NS3 activated MazF or the red fluorescent protein alone, suggesting that expression of NS3-activable ribonuclease in naïve HEK293 T-REx cells (that do not express NS3) cause minimal toxicity, if any. As expected, growth was severely inhibited when cells were transfected with the EGFP-fused active (uninhibited) toxin.

### The ER membrane-targeted zymoxin colocalizes with NS3 protease *in vivo*

**Figure 3:**
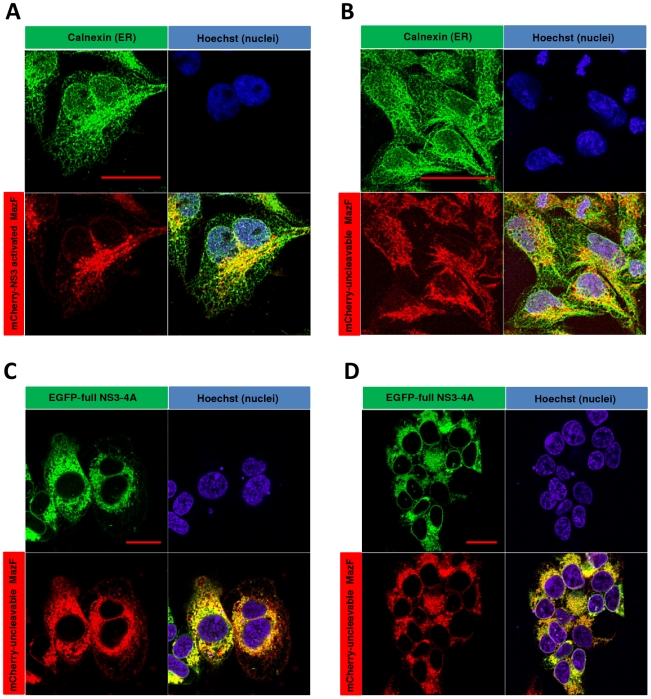
Cellular colocalization of NS3 protease and the ER-membrane targeted MazF based zymoxin. 1×10^5^ Tet-NS3/activated MazF or Tet-NS3/uncleavable MazF cells were seeded on poly-L-lysine coated cover-slips in a 24 well-plate. 12 h later, the cells were supplemented with 1 µg/ml of tetracycline or left untreated. After 24 h, the cells were fixed. Uninduced Tet-NS3/activated (A) and uncleavable (B) MazF cells were permeabilized and incubated with rabbit-polyclonal anti-calnexin antibody followed by Cy2-conjugated anti-rabbit IgG secondary antibody for ER visualization (green). Nuclei of the uninduced cells (A and B) and induced Tet-NS3/uncleavable MaF cells (C and D) were stained by Hoechst 33258 (blue). Slides were then mounted and examined by confocal fluorescence microscope. The bar represents 20 µm.

Previously we described a HEK293 cell line which inducibly expresses (by addition of tetracycline) a fusion between EGFP and the coding sequence of the full length NS3 (including the helicase domain) followed by NS4A from HCV 1a genotype [10]. These cells, denoted “Tet-inducible full NS3-4A expressing cells”, were stably transfected with a plasmid encoding the NS3-activated zymoxin “mCherry-NS3 activated MazF” or with the uncleavable control (“mCherry-uncleavable MazF”) encoding plasmid. Following selection, stable clones that constitutively express high level of the cleavable construct (denoted “Tet-NS3/activated MazF cells”) or the uncleavable control (denoted “Tet-NS3/uncleavable MazF cells”) were isolated. In order to characterize the intracellular distribution of the mCherry fused zymoxins, Tet-NS3/activated or uncleavable MazF cells were subjected to immunofluorescence microscopy analysis. As shown in **Figure 3** (**A and B**), both zymoxins have a similar cellular distribution, colocalizing with the ER marker calnexin at the juxtanuclear region of the ER. Importantly, upon induced expression of NS3-A4 in Tet-NS3/uncleavable MazF cells, a colocalization of the two fluorescent fusion proteins could be observed (**Figure 3, C and D**).These observations confirm that indeed both the protease and the modified ribonuclease are tethered to a common cellular compartment, presumably the cytoplasmic side of the ER membrane (**see scheme in** **Figure 1**).

### NS3-mediated proteolytic activation of MazF based zymoxin inhibits *de-novo* cellular protein synthesis

**Figure 4:**
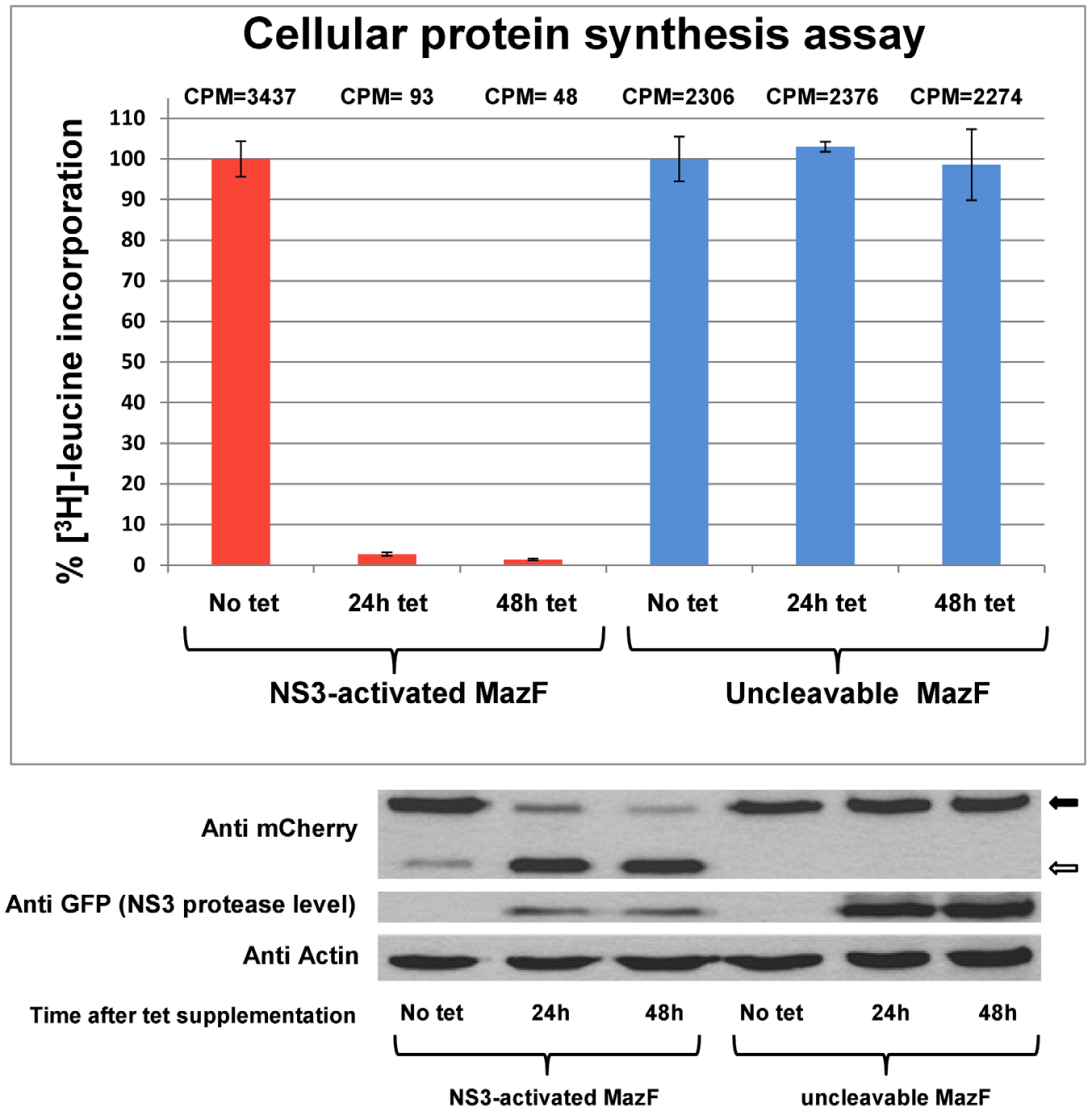
Inhibition of *de-novo* protein synthesis by NS3-activated MazF based zymoxin in NS3-expressing cells. 1×10^5^ Tet-NS3/activated MazF or Tet-NS3/uncleavable MazF cells were seeded per well in 24-wells plate. 24 or 48 h later, cells were supplemented with tetracycline to a final concentration of 1000 ng/ml, or left untreated (48 h tet, 24 h tet and no tet, respectively). 72 h after seeding, levels of *de-novo* protein synthesis were determined by [^3^H]-leucine incorporation assay, as described in “materials and methods”. Results are expressed as percent of the value obtained for cells which were not induced to express the NS3 protease (No tet). Each bar represents the mean ± SD of a set of data determined in triplicates. Numbers above each bar represent mean counts per minute (CPM) values for 7 micrograms total protein samples (upper panel). 30 micrograms of total protein from lysates of the described cells were analyzed by immunoblotting with mouse anti-mCherry (for detection of the zymoxin), mouse anti-GFP (for the detection of EGFP-NS3) and mouse anti actin antibodies (loading control) followed by HRP-conjugated secondary antibodies and ECL development. Solid arrow: full length zymoxin. Hollow arrow: N' terminal portion of NS3-cleaved zymoxin (lower panel).

Since expression of active MazF was found to inhibit *de-novo* protein synthesis in mammalian cells [27], we hypothesized that such an effect may be observed also in cells in which MazF based zymoxin is proteolytically activated. In order to validate this assumption, Tet-NS3/activated MazF and Tet-NS3/uncleavable MazF cells were supplemented with tetracycline for 24 or 48 h, or left untreated. Levels of *de-novo* protein synthesis were than determined by [^3^H]-leucine incorporation assay. As shown in **Figure 4** **(upper panel)**, a complete shutoff in protein synthesis was observed as soon as 24 h post NS3 induction in cells that express the cleavable construct, indicating proteolytic activation of the zymoxin. As expected, protein synthesis was not impaired following NS3 induction in cells that express the uncleavable toxin.

In support of these findings, an immunoblot assay revealed a near complete cleavage of the zymoxin following tetracycline-induced expression of NS3 protease in NS3-activated MazF expressing cells (**Figure 4** **, lower panel**). Presumably, this proteolytic activation of the ribonuclease toxin resulted in a cessation of cellular protein synthesis soon after induction, what explains the detection of relatively low levels of NS3 protease in these cells. As expected, no zymoxin cleavage could be detected in cells expressing the uncleavable form of the toxin. A faint band, corresponding to a cleaved zymoxin, can also be detected in uninduced cells that express the NS3 cleavable construct. This cleavage can be attributed to the “leakiness” of the Tet inducible system, allowing a very low basal transcription of the protease in the absence of externally added tetracycline. Such cleavage could not be detected in “naïve” cells following transfection with NS3-cleavage substrate encoding transgene ([10], data not shown). Apparently, this very low, basal proteolytic activity is well tolerated by NS3 activated zymoxin expressing cells that shows no indication of *de-novo* protein synthesis inhibition in comparison to cells that express the uncleavable toxin (see CPM values in the upper panel of **Figure 4**).

### NS3-activated MazF eradicates cells that express the NS3 protease

**Figure 5:**
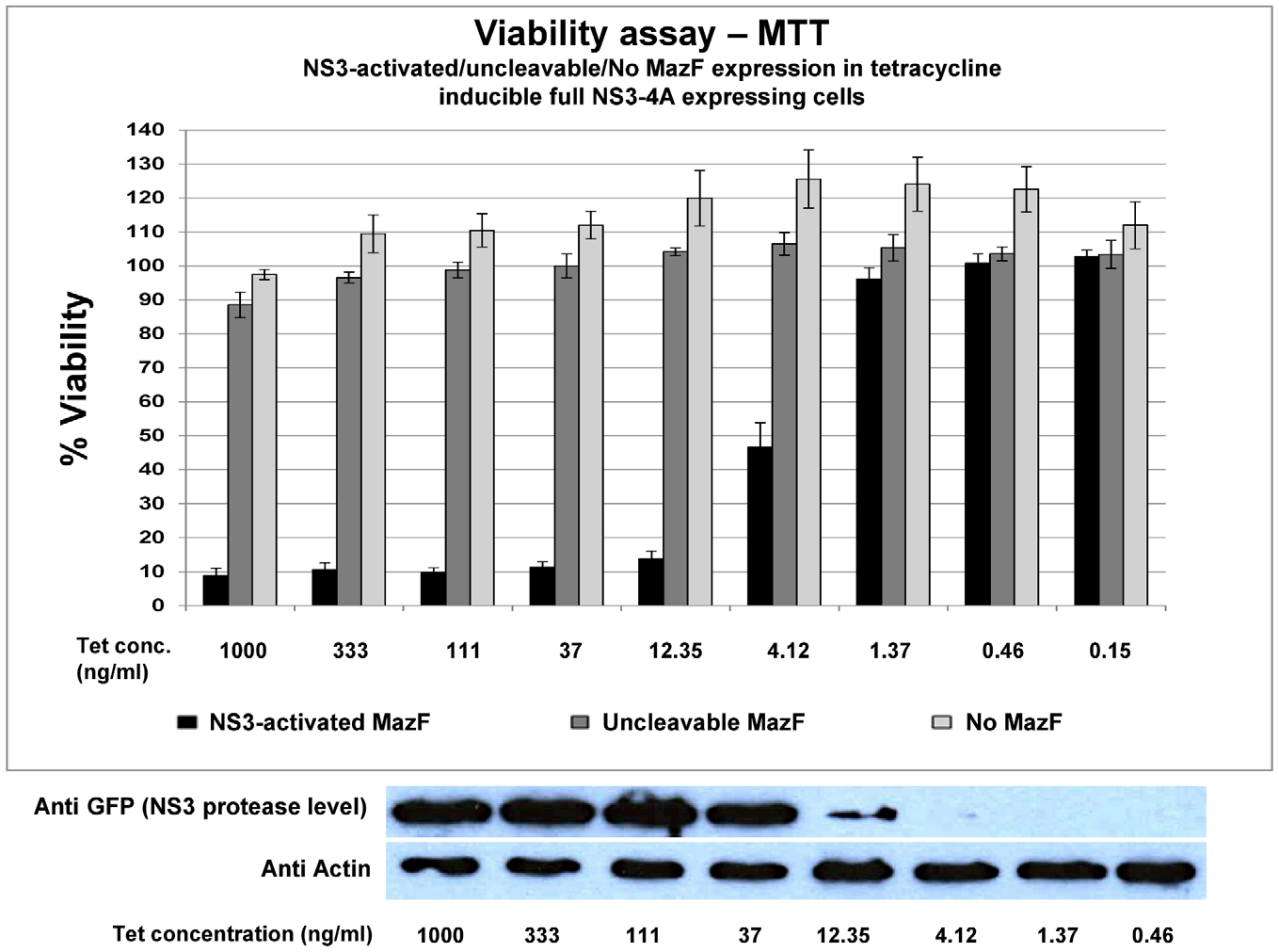
Eradication of NS3 expressing cells by mCherry-NS3 activated MazF. Upper panel: Tet-inducible full NS3-4A (No MazF), Tet-NS3/activated MazF (NS3-activated MazF) or Tet-NS3/uncleavable MazF (uncleavable MazF) cells were seeded in 96 well plates (2×10^4^ cells per well). After 24 h, cells were supplemented with 3 fold dilutions of tetracycline starting with concentration of 1000 ng/ml, or left untreated. 72 hours later, the fraction of viable cells (relatively to the untreated controls) was determined using an enzymatic MTT assay. Each bar represents the mean ±SD of a set of data from six wells. Lower panel: 30 ng of total protein from lysates of Tet-NS3/uncleavable MazF cells that were supplemented with 3 fold dilutions of tetracycline for 48 h were analyzed by immunoblotting with mouse anti-GFP (for the detection of EGFP-NS3) and mouse anti-actin antibodies (loading control) followed by HRP-conjugated secondary antibodies and ECL development.

**Figure 6:**
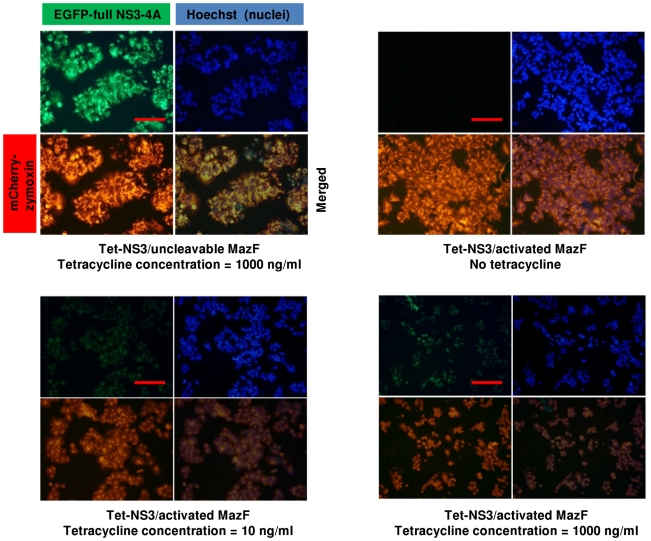
Expression of mCherry-NS3 activated MazF results in growth inhibition and morphological changes in NS3-expressing cells. 1×10^5^ Tet-NS3/activated MazF or Tet-NS3/uncleavable MazF cells were seeded on poly-L-lysine coated cover-slips in a 24 well-plate. 12 h later, cells were supplemented with 10 ng/ml or 1000 ng/ml of tetracycline, or left untreated. 36 h later, cells were fixed. Following nuclear staining by Hoechst 33258 (Blue), slides were examined by fluorescence microscopy. The bar represents 50 µm.

To evaluate the potential of the NS3-cleavable MazF based zymoxin in eradication of NS3 expressing cells, three 96 well plates were seeded with Tet-inducible full NS3-4A, Tet-NS3/activated MazF or Tet-NS3/uncleavable MazF cells and were supplemented with 3 fold serial dilutions of tetracycline starting with 1000 ng/ml. After 72 h, the relative fraction of viable cells was determined using an enzymatic MTT assay. As shown in **Figure 5** **(lower panel)**, the expression level of NS3 can be roughly tuned by modulation of the final tetracycline concentration in the growth media, with around 10 ng/ml as an intermediate concentration for induction of low NS3 expression level. Indeed, strong cytotoxicity was clearly evident when Tet-NS3/activated MazF cells where treated with tetracycline concentrations of down to 4 ng/ml. No cytotoxic effect was detected when the controls Tet-NS3/uncleavable MazF or Tet-inducible full NS3-4A (protease only) expressing cells were similarly treated (**Figure 5** **, upper panel**). These findings demonstrate the deleterious effect of the MazF based zymoxin specifically toward NS3 protease expressing cells, as well as its competence to be activated by very low cellular levels of protease. In order to obtain a visual insight at the cell level, Tet-NS3/activated MazF and Tet-NS3/uncleavable MazF cells were supplemented with tetracycline to a final concentration of 10 ng/ml or 1000 ng/ml (for low and high induction levels of NS3 expression, respectively), or left untreated. 36 h later, nuclei were stained and cells were examined under a fluorescence microscope. The results show that both lower and higher induction levels of NS3 protease caused growth inhibition and rounding of cells that constitutively expresses the cleavable MazF. Furthermore, both green and red fluorescence were faint in these cells, probably as a result of the destructive ribonuclease activity of the cleaved toxin toward the NS3 protease and its own mRNA. As expected, none of the above observations was evident when these cells were not supplemented with tetracycline or when NS3 expression was induced to high level in cells that constitutively express the uncleavable toxin (**Figure 6**).

### Adenovirus-mediated delivery of mCherry-NS3 activated MazF encoding cassette specifically eradicates NS3 expressing hepatocytes

To achieve efficient DNA delivery into mammalian cells, the expression cassette encoding mCherry-NS3 activated MazF was cloned into a “first generation” ΔE1/ΔE3 human type 5 adenoviral vector plasmid DNA by homologous recombination in bacteria [35]. Virus particles were propagated in HEK293 packaging cells as described in the “materials and methods” section. In addition, a control adenoviral vector was constructed for the delivery of a similar cassette that encodes for the uncleavable version of the construct (mCherry-uncleavable MazF). Red-fluorescent comet-like adenovirus-producing foci were apparent upon infection of packaging cells with both recombinant viruses (encoding cleavable or uncleavable constructs) (see **Figure S1**). The production yields for both viruses were ∼3×10^8^ plaque forming units (PFU)/ml, after two “cycles” of virus amplification (see **Text S2**).

In order to evaluate the ability of adenovirus-mediated delivery of NS3 activated MazF encoding cassette to eradicate NS3 expressing hepatocytes, wild-type Huh7.5 hepatoma cells and our previously described EGFP-full NS3-4A expressing Huh7.5 cells [10] were infected with the NS3-activated or uncleavable MazF encoding viruses. Since our previous unpublished observations and studies of others have indicated that high level of transgene expression and adenoviral infection, *per se*, may adversely affect cell viability and growth [36], [37], [38], [39]; the cells were infected with a series of multiplicity of infection (MOI) ratios in order to find an optimal MOI that leads to eradication of NS3-expressing cells while maintaining minimal toxicity to naïve cells.

**Figure 7:**
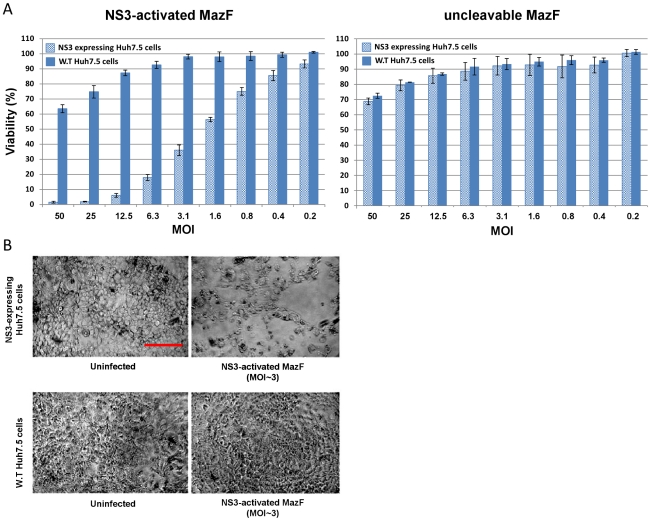
Eradication of NS3-expressing Huh7.5 cells by recombinant adenovirus-mediated delivery of mCherry-NS3 activated MazF encoding cassette. 1×10^4^ wild-type (W.T) or EGFP-full NS3-4A expressing Huh7.5 cells were seeded per well in 96 well plates. After 24 h, recombinant adenoviruses encoding for mCherry-fused NS3 activated MazF or uncleavable-MazF zymoxins were added at the indicated MOI's. Control cells remained untreated. (A) MTT viability assay: 4 days post infection, the fraction of viable cells (relatively to uninfected controls) was determined using an enzymatic MTT assay. A representative graph of three independent experiments is shown. Each bar represents the mean ±SD of a set of data determined in triplicates. (B) Microscopic examination: 4 days post infection, wild-type (lower panel) or EGFP-full NS3-4A expressing Huh7.5 cells (upper panel), uninfected or infected with recombinant adenoviruses encoding for mCherry fused NS3-activated MazF zymoxin (at MOI of ∼3), were fixed and subjected to microscopic examination. The bar represents 200 µm.

As shown in **Figure 7A** **(left panel)**, infection with the NS3-activated zymoxin resulted in a considerable cytotoxic effect against NS3-expressing hepatocytes, leading to their almost complete eradication at MOI's≥12. However, infection at these ratios also adversely affected the growth of wild-type hepatocytes. In contrast, infection at MOI of ∼3 decreased the viability of the NS3-expressing Huh7.5 cells to about 35% of the untreated control without affecting the viability of the wild-type hepatocytes (see also microscopic examination in **Figure 7B**). Therefore, infection at this MOI was applied during the next experiments. As expected, no substantial enhancement in cytotoxicity against NS3 expressing Huh7.5 cells (relatively to wild-type Huh7.5 cells) was observed, at any MOI, upon infection with the uncleavable MazF encoding viruses (**Figure 7A** **, right panel**).

### Adenovirus-mediated delivery of NS3-activated MazF encoding cassette specifically eradicates HCV infected hepatocytes

**Figure 8:**
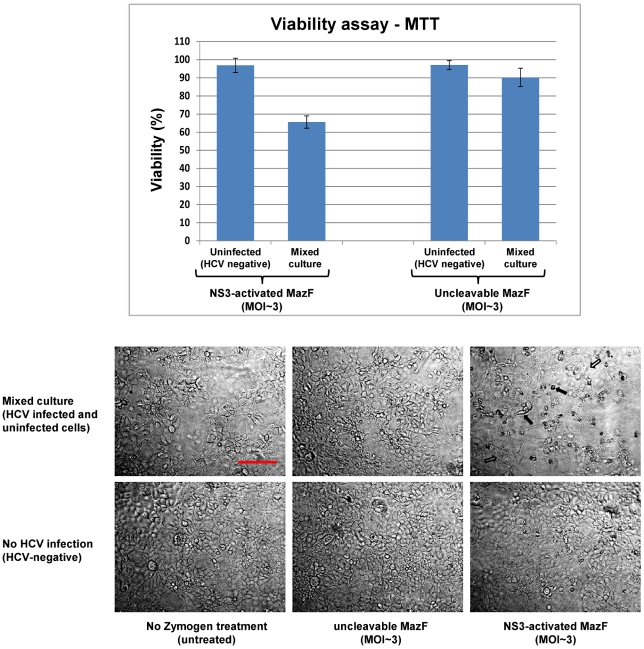
Treatment of HCV infected/uninfected mixed cell culture with recombinant adenovirus-delivered MazF based zymoxin. Uninfected (HCV-negative) Huh7.5 cells and a mixed culture of HCV infected and uninfected cells at 1∶1 ratio (50% infected culture) were seeded in 96-well plates (1×10^4^ cells/well). After 24 h, cells were treated with recombinant adenoviruses (MOI of ∼3) encoding for the mCherry fused NS3-activated MazF or uncleavable-MazF zymoxins. Control cells remained untreated. Upper panel: MTT viability assay: 72 h post treatment, the fraction of viable cells (relatively to untreated controls) was determined using an enzymatic MTT assay. A representative graph of three independent experiments is shown. Each bar represents the mean ±SD of a set of data determined in triplicates. Lower panel: Microscopic examination: 72 h post treatment, the uninfected (HCV-negative) Huh7.5 cells, the mixed culture of HCV infected and uninfected cells and the control untreated cells were fixed and subjected to microscopic examination. Hollow arrows point to cells that are characterized by a “typical” Huh7.5 cell morphology. Filled arrows point to partially detached cells with round, condensed or distorted shape. The bar represents 200 µm.

**Figure 9:**
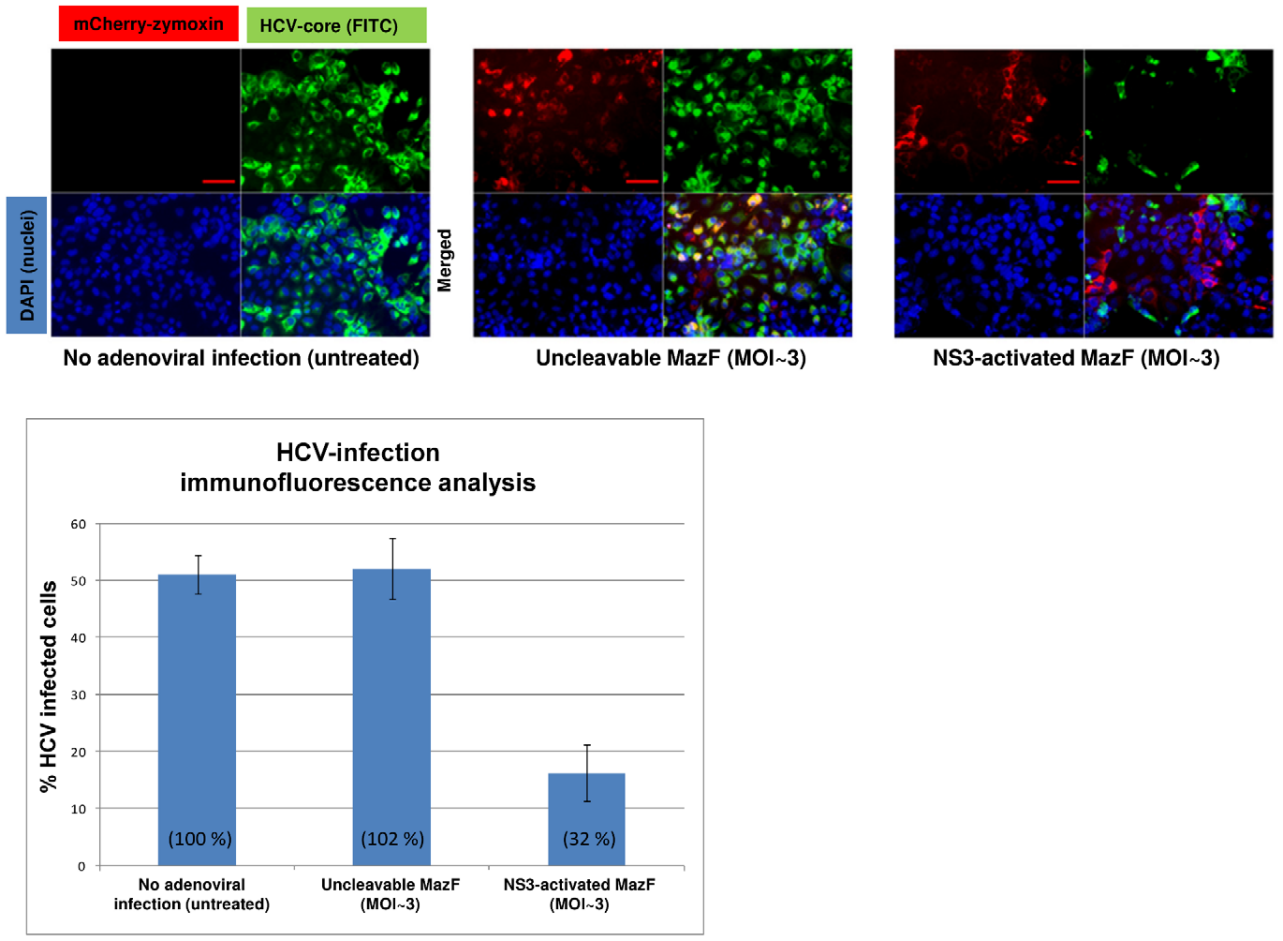
Eradication of HCV-infected hepatocytes by recombinant-adenovirus delivered MazF based zymoxin. 3×10^4^ cells from mixed HCV infected and uninfected culture (at 1∶1 ratio) were seeded per well into 8-well chamber slides. 24 h later, cells were treated with recombinant adenoviruses (MOI of ∼3) encoding for the mCherry fused NS3-activated MazF or uncleavable-MazF zymoxins. Control cells remained untreated. 72 h post treatment, cells were fixed, permeabilized and immunostained with mouse anti-HCV core and FITC-conjugated goat anti mouse antibodies for visualization of infected cells (green). Cell nuclei were then stained with DAPI (cyan) and slides were visualized by fluorescence microscopy. The bar represents 100 µm (upper panel). The fraction (given in percentage) of HCV-infected cells from the general cell population was evaluated, for each treatment, by dividing the number of the green, HCV-core positive cells by the general number of cells (DAPI stained) (lower panel). Each bar represents the mean ±SD of a set of data collected from five representative microscopic fields. Numbers in brackets represent the percentage of the HCV-infected cells in each treatment relatively to their percentage in the untreated culture.

To test the competence of the MazF based zymoxin to specifically eradicate HCV infected hepatocytes, we utilized the infectious chimeric virus HJ3-5 [40]. This is one of the currently used models to study hepatitis C virus in which recombinant infectious HCV particles are produced in cell culture (HCVcc) (for review, see [41], [42]). As the main purpose of zymoxins based treatment is the specific eradication of HCV infected cells from a background of healthy cells, “mixed culture” experiments were carried out. In these experiments, Huh7.5 hepatoma cells were infected with the HCV 1a/2a chimeric virus HJ3-5 (encoding the NS3 protease of genotype 2a strain JFH1 [40]). When infection reached ∼50% (about 50% of the cultured cells showed expression of the HCV-core protein, as detected by immunostaining and fluorescence microscopy), the mixed culture and a culture of uninfected cells were treated with NS3 activated MazF or uncleavable-MazF encoding adenoviruses at MOI of ∼3. Control cells remained untreated. 72 h post adenoviral infection; viability assay and microscopic examination that included immunostaining for HCV-core protein were performed. As shown, treating the mixed culture with NS3-activated MazF zymoxin-encoding adenovirus reduced the viable cells population to about 65% relatively to untreated control; while viability of the uninfected cells (“HCV negative”) was barely affected by this treatment (**Figure 8** **, upper panel**). Microscopic examination of the treated mixed culture revealed two cell populations that differ in their appearance. While one population is characterized by a “typical” Huh7.5 cell morphology (hollow arrows, **Figure 8** **, lower panel**), the other is composed of partially detached cells with round, condensed or distorted shape (filled arrows) that are hypothesized to be zymoxin-intoxicated HCV infected cells. In order to validate this assumption, the fraction of HCV infected cells from the general population was evaluated by immunofluorescence analysis using anti-HCV core protein specific antibodies. Indeed, treatment with the NS3-activated zymoxin showed a “curing effect” upon the partially infected culture, considerably reducing the fraction of the HCV infected cells from the general population (**Figure 9**). As expected, no significant effect upon the HCV infected cell population was observed following treatment with the uncleavable zymoxin.

## Discussion

Fight against viral infections is considered as one of the most challenging areas in modern medicine. While vaccination is considered to be the most efficient method for fighting viral infections; for some viral pathogens which cause world-wide health problem like human immunodeficiency virus (HIV) and hepatitis C virus (HCV), no efficient vaccine has yet been developed. Over the last years, efforts have been focused on the discovery and development of anti-viral agents that target crucial steps in the viral replication cycle which includes viral entry, RNA translation and post-translational processing, reverse transcription, genome integration, viral assembly and release [43], [44], [45].

An essential step in the replication cycle of many viruses is the processing of a polyprotein precursor by a viral-encoded protease. A partial list of human diseases associated viruses encoding protease(s) in their genome include flaviviruses such as hepatitis C virus (HCV), West Nile virus (WNV), dengue fever virus (DFV) and yellow fever virus (YFV); retroviruses such as HIV-1; picornaviruses such as coxsackievirus, poliovirus and hepatitis A virus; nidoviruses such as coronaviruses (CoV), including the severe acute respiratory syndrome (SARS) causative SARS-CoV and herpesviruses such as varicella-zoster virus (VZV) and Epstein-Bar virus (EBV) [46], [47]. Therefore, a large part of antiviral drug discovery is focused on inhibiting viral proteases [46]. Our group too has published several studies where HCV replication was inhibited by intracellular expression of antibodies and peptide aptamers [48], [49], [50]. The opposite “side of the coin” – taking advantage of the activity of a viral protease *per-se* as an antiviral approach has been rather neglected.

Recently we described a study that was based on the concept of “sitoxins” which are anti-viral agents that are designed to eradicate viral-infected cells by taking advantage of a specific viral activity instead of inhibiting it [51], [52]. specifically, a sitoxin is comprised of an effector domain (e.g. a toxin); a domain bearing an intracellular signaling moiety (e.g. a degradation or an intracellular localization signal); and a domain located between the effector domain and the domain bearing the intracellular signaling moiety which specifies a cleavage site for a predetermined protease (e.g. a viral encoded protease). Following the introduction of the sitoxin into the target cell (that expresses the specific protease), cleavage by the predetermined protease separates the toxic effector domain of the sitoxin from the intracellular signaling moiety, resulting either in a longer-lived (and therefore more toxic) effector domain or in an effector domain that moves from a cellular compartment where the domain is nontoxic to a cellular compartment where the domain is able to exert its effect [51]. We developed and successfully evaluated two rationally designed viral-protease-activated chimeric toxins which we named “zymoxins” for “zymogenized toxins” [10]. In contrast to sitoxins, in which the inhibitory activity is mediated by intracellular components which recognize a cleavable signaling polypeptide fused to a constitutively active toxin; the concept of zymoxins is based on the idea of reengineering a toxic enzyme into an inactive zymogen which is specifically activated by a predetermined protease. This general approach was demonstrated by other elegant studies in which several enzymes, including bovine RNase A, Vip2 and the maize ribosome inactivating protein (maize-RIP) were converted into protease activated forms [53], [54], [55], [56], [57].

Our previous work focused on the design of protein-delivered toxins that were converted into protease-activated forms by means of fusion to specific, rationally designed inhibitory peptides through HCV-NS3 protease cleavable linker. When tested *in-vitro* and on NS3-overexpressing or HCV infected cells, a clear NS3 protease cleavage-dependent enhancement in activity/cytotoxicity was observed. However, these constructs had two major drawbacks, as mentioned in the introduction: The first is the incomplete inhibition of the toxic enzymatic activity that is conferred by the rationally designed fused peptide. The second relates to the necessity of relatively high level of expressed viral protease for achieving adequate zymoxin activation inside the cells [10].

In the current study, we turned to evaluate a similar strategy for eradicating HCV-infected cells, namely, by converting a constitutively active enzyme into an NS3 activated zymogen. As a toxic moiety, we chose MazF, an endoribonuclease that together with its polypeptidic antidote, MazE, constitute one of the most studied “genomic” toxin-antitoxin systems in *E. coli*. In order to convert MazF into a zymoxin, a fusion polypeptide was constructed in which an inhibitory peptide derived from the MazE antitoxin was fused to the C terminus of the MazF toxin via an NS3-cleavable linker. Regarding the issue of incomplete inhibition of zymoxin's activity in uncleaved form, it should be mentioned that in contrast to our previously described constructs, in which a “rationally designed” peptide is appended to provide the inhibition of the toxin's activity; in the MazF based-zymoxin, a “natural” inhibitory polypeptide was chosen for that purpose. Antidote polypeptides in toxin-antitoxin systems were “evolutionary shaped” to strongly inhibit the destructive activity of their toxic counterparts. Thus, a very efficient inhibition may be achieved when using them as inhibitory peptides in the construction of zymoxins. Indeed, our results show that the fused MazE-derived inhibitory peptide diminishes the toxin's activity to such an extent that enables its non-lethal overexpression in naïve cells.

In order to improve the responsiveness of the zymoxin to the presence of low levels of cellular-expressed viral protease, an ER membrane “anchoring peptide” was fused to the C terminus of the construct, subsequently to the MazE derived inhibitory peptide. By using that design, a colocalization between the ER-bound NS3 protease and its zymoxin substrate might be achieved, resulting in an improved cleavage efficiency. Indeed, such a colocalization could be observed, as shown above (**Figure 3**). Moreover, activation of the zymoxin was evident also upon expression of very low cellular levels of the viral protease (**Figures 5** **and** **6**).

It should also be mentioned that while being extremely sensitive to minute amounts of expressed NS3 protease, zymoxin expressing cells can still tolerate some degree of activating proteolysis (as shown above for NS3-activated MazF expressing cells that show a very low, basal NS3 proteolytic activity without tetracycline induction (**Figure 4**)). This property may, in fact, contribute to the specificity and general safety of zymoxin-based therapeutics, even though “spontaneous”, unspecific proteolytic activity toward NS3 substrates could not be detected in naïve cells during our experiments (data not shown). As discussed, tethering the zymoxin to the ER membrane through its inhibitory peptide may also enhance its cleavage-dependent toxicity. This might be achieved by enabling spatial separation between the activated toxin and its antidote following NS3-cleavage and diffusion of the active MazF to the inhibitory peptide-free cytoplasm. Supporting this assumption, we found that although cleaved, a cytoplasmic version of the NS3-activated MazF based zymoxin is barely toxic to NS3 protease-expressing cells (data not shown).

In contrast to our previous study, in which the toxins were delivered into mammalian cells as purified recombinant proteins; in the current research an adenoviral vector gene-delivery system was used. Widely applied in gene therapy, recombinant viral vaccines and basic science studies, this system enables efficient delivery and high level of transgene expression in a wide variety of human cells. Using this system, we have demonstrated specific eradication of full NS3-4A expressing Huh7.5 cells, sparing “naïve” cells which do not express the protease. Furthermore, specific eradication of HCV infected cells has also been achieved, demonstrating a prominent “curing effect” when tested on mixed cultures of healthy and HCV infected hepatocytes.

The use of viral gene delivery for treating HCV infection by eradication of infected cells has been proved successful in previous study on mice with chimeric human livers. In that study, Hsu *et al* designed a modified proapoptotic BID molecule in which its endogenous cleavage sites were replaced by NS3 recognition site. As BID requires proteolytic processing in order to activate its apoptotic function, infection with adenoviral vector that delivers a transgene encoding the engineered BID molecule was demonstrated to induce activation of apoptosis in cells expressing the HCV NS3 protease. Moreover, a significant reduction in HCV titer in the serum of the HCV infected mice was detected following injection of the engineered adenoviral vector into the jugular vein [58].

Although such an approach had proved successful; it should be taken into account that a delivered transgene which encodes for a modified form of a protein that is naturally expressed in the target cell has the potential to negatively influence normal cellular processes in which its endogenous counterpart plays a role [59], [60], [61], [62], [63]. For example, when overexpressed in non-infected cells, an NS3-cleavable apoptotic molecule, such as that described above, may act as a “dominant negative” by competing with the natural, endogenous protein for interactions with activators, regulators or substrates. Delivery of a transgene encoding for a foreign protein which has no endogenous counterpart in target cells, such as MazF, may reduce that risk. Furthermore, it should be noted that MazF possess a ribonuclease activity that is also capable of processing multiple potential cleavage sites in the HCV genome. Although not evaluated in this study, a direct attack on the parasite's genetic material may represent an additional mode of anti-viral activity that works in parallel with host-cell protein synthesis shutoff.

Of note, the applicability of the described zymoxin may be extended by replacing the protease cleavage site that separates between the toxic moiety and the inhibitory peptide. This could facilitate eradication of cells that are infected with other protease expressing viruses other than HCV (a partial list of protease expressing viruses is given at the beginning of the discussion) or different strains of HCV that differ in their NS3 protease cleavage specificity. In addition, one may replace the C terminal ER anchoring peptide with sequence that tethers the construct to a different intracellular location in which the viral protease resides. In that way, a colocalization between the zymoxin and the predetermined viral protease may be achieved.

In conclusion, the presented anti-viral agent was designed following our previously described “zymoxins” concept in which a constitutively active toxin is converted into a “zymogenized”, viral-protease activated from. The MazF-based zymoxin, that is introduced to target cells by means of adenovirus mediated gene delivery, shows very low toxicity to naïve cells and enhanced responsiveness to low viral-protease expression level, when compared to our previously presented constructs. As evident from our results, the MazF based zymoxin eradicates NS3-expressing model cells and HCV infected cells with remarkable efficiency and specificity, providing further proof to the concept of zymoxins and a potential new means of fighting viral diseases. Obviously, further optimizations and pharmacological assessments in animal models may be required in order to determine the safety and efficiency of zymoxins treatment in the context of the whole organism.

## Materials and Methods

### Bacterial strains

The following *Escherichia coli* (*E. coli*) strains were used: XL-1 Blue and DH5α (Stratagene, USA) for plasmid propagation and BJ5183 (Stratagene, USA) for the generation of recombinant adenovirus plasmid DNA.

### Recombinant DNA techniques and vectors

Recombinant DNA techniques were carried out according to standard protocols or as recommended by the suppliers. Nucleotide sequences were determined using the PRISM 3100 Genetic Analyzer (Applied Biosystems, USA) according to the supplier's recommendations. The eukaryotic CMV promoter-based GFP-fusion expression vector pEGFP C2, which was used for expression of mCherry, MazF and MazF-based zymoxins, was from Clontech (USA). The AdEasy plasmid system (pShuttle and pAdEasy-1) [35], that was used for generation of recombinant human type 5 adenoviral vectors for gene delivery of the zymoxins expression cassettes, was a generous gift from Prof. Nadir Arber, Integrated Cancer Prevention Center, Tel Aviv Sourasky Medical Center, Israel. All plasmid and DNA fragment purifications were carried out with High-Speed Plasmid Mini Kit and Gel/PCR DNA fragments Extraction Kit (Geneaid Biotech Ltd., Taiwan) unless mentioned otherwise. T4 DNA ligase and restriction enzymes were purchased from New England Biolabs (USA). DNA ligations were carried out at 16°C overnight.

### Molecular cloning

#### Oligonucleotides

All the oligonucleotides that were used in this study were purchased from Hylabs, Israel. Oligonucleotides that were used in this study are listed in **Table S1**.

#### Construction of the vector encoding for “mCherry-NS3 activated MazF”

A polymerase chain reaction (PCR) was carried out using a single colony of *Escherichia coli* strain XL-1 as a template, the forward primer: 40- clvmazf and the reverse primers: 41-clvmazf, 42-clvmazf, 43-clvmazf, 44-clvmazf, 45-clvmazf, 46-clvmazf and 47-clvmazf. The PCR product, encoding for a fusion polypeptide composed of (from the N terminus) MazF, HCV P10-P10' NS3 cleavage sequence derived from 2a genotype (strain JFH1) NS5A/B junction, a short flexible linker, a short inhibitory peptide corresponding to MazE C-terminal 35 amino-acids (which encompass the 23 amino-acids inhibitory peptide (MazEp) that has been described by Li *et al.* [25]), a flexible linker and the C-terminal ER membrane anchor of the tyrosine phosphatase PTP1B [28] was digested with *Xho*I and *Eco*RI and was cloned between the corresponding sites in the plasmid pEGFP-C2, generating plasmid “pEGFP-NS3 activated MazF”. Next, the sequence of the red fluorescent protein mCherry [29] was amplified by PCR from an expression cassette (kindly provided by Prof. Adi Avni, Department of Molecular Biology and Ecology of Plants, Tel-Aviv university, Israel) using the forward primer: 48- clvmazf and the reverse primer: 49- clvmazf. The PCR product was digested with *Nhe*I and *Xho*I and was cloned between the corresponding sites of the plasmid “pEGFP-NS3 activated MazF” (replacing the EGFP coding sequence), generating plasmid: “pmCherry-NS3 activated MazF”. The amino-acid sequence of the encoded cleavable zymoxin can be found in **Text S1**.

#### Construction of the vector encoding for “mCherry-uncleavable MazF”

A PCR was carried out using DNA of plasmid “pmCherry-NS3 activated MazF” as a template, the forward primer: 40- clvmazf and the reverse primers: 50-unclmazf and 51-unclmazf. The PCR product was digested with *Eco*RV and *Nru*I, and the digestion product of 233 bp was cloned between the corresponding sites of the same plasmid that has been used as a template, generating the plasmid: “pmCherry–uncleavable MazF”. This plasmid encodes for an uncleavable construct in which the NS3 cleavage sequence was replaced by a mutated 14 amino acids cleavage sequence (P10-P4') from HCV genotype 1a NS5A/B junction in which P3 valine was substituted by alanine, P2 cysteine by glycine, P1 cysteine by glycine and P4' tyrosine by alanine. The amino-acid sequence of the encoded uncleavable zymoxin can be found in **Text S1**.

#### Construction of the vector encoding for “EGFP-MazF”

This is a mutated variant of an “intermediate” vector used in the construction process of the “mCherry-NS3 activated MazF” encoding vector. In this variant, a nonsense mutation was inserted instead of the tyrosine in the SMSY sequence of the NS3 recognition site, generating the plasmid “pEGFP-MazF” that encodes for a truncated EGFP-MazF fusion protein that lacks the MazE derived inhibitory peptide and the ER anchor.

#### Construction of the vector encoding for mCherry

The sequence of the red fluorescent protein mCherry [29] was amplified by PCR from an expression cassette (see construction of the vector encoding for “mCherry-NS3 activated MazF”) using the forward primer: 48- clvmazf and the reverse primer: 49- clvmazf. The PCR product was digested with *Nhe*I and *Bgl*II and was cloned between the corresponding sites of the plasmid pEGFP C2 (replacing the EGFP coding sequence), generating the plasmid “pmCherry”.

#### Construction and propagation of recombinant adenoviral vectors

Construction and propagation of recombinant human type 5 adenoviral vectors for gene delivery of the mCherry-NS3 activated MazF and mCherry-uncleavable MazF expression cassettes was carried out using the AdEasy system essentially as described in [35], [64]. A more detailed description of the procedure is provided in **Text S2**.

### Cell culture, transfection, protein extraction and immunoblotting

Human embryonic kidney cells HEK293, stably expressing the tetracycline repressor protein (T-REx HEK293 Cell Line, Invitrogen, USA), and human hepatoma cells Huh7.5 [65] were used throughout this study. Cell lines were maintained in Dulbecco's Modified Eagle Medium (DMEM) supplemented with 10% fetal calf serum (FCS), 2 mM L-glutamine, 100 U/ml penicillin and 100 µg/ml streptomycin (Biological Industries, Israel) in a humidified 5% CO_2_ incubator at 37°C.

The calcium-phosphate transfection method was applied for introducing 2 µg of the plasmid “pmCherry-NS3 activated MazF” or the plasmid “pmCherry-uncleavable MazF” into T-REx HEK293 cells inducibly expressing EGFP-Full NS3-4A [10] seeded 1.5×10^6^ cells per 60 mm plate 24 h before transfection. Stable transfectants, inducibly expressing EGFP-Full NS3-4A and constitutively expressing mCherry-NS3 activated MazF (denoted “Tet-NS3/activated MazF cells”) or mCherry-uncleavable MazF (denoted “Tet-NS3/uncleavable MazF cells”) were selected in a medium containing 1 mg/ml of G418 (A.G. Scientific, USA). Cell clones that express high level of the cleavable construct or the uncleavable control were identified by fluorescence microscopy and isolated.

For protein extraction, 48 h post-transfection the cells were washed with PBS, scraped and lysed in a buffer containing 150 mM NaCl, 5 mM EDTA, 0.5% NP-40, 10 mM Tris(HCl) pH 7.5, and protease inhibitors cocktail (Sigma, Israel). Following 30 minutes of incubation on ice, lysates were cleared by centrifugation at 20,000 *g* for 10 minutes, at 4°C. For immunoblotting, protein samples were electrophoresed on 12% SDS/polyacrylamide gel, transferred to nitrocellulose and detected using mouse monoclonal anti-mCherry antibody (Clontech, USA), mouse monoclonal anti-GFP antibody (Santa-Cruz, USA) or mouse monoclonal anti-actin antibody (Abcam, USA), followed by horseradish peroxidase (HRP)-conjugated goat anti-mouse antibodies (Jackson ImmunoResearch Laboratories, USA) and enhanced chemiluminescence (ECL) detection using SuperSignal West Pico Chemiluminescent Substrate (Thermo SCIENTIFIC/Pierce, USA).

### HCV infection

Virus assays were carried out with an inter-genotypic chimeric hepatitis C virus (HCV) produced by replacing the core-NS2 segment of the JFH-1 virus genome with the comparable segment of the genotype 1a H77 virus. This chimeric virus, HJ3-5 (Kindly provided by Prof. Stanley Lemon, University of Texas at Galveston), contains two compensatory mutations that promote its growth in cell culture as described previously [40]. HCV RNAs were transcribed *in vitro* and electroporated into cells essentially as described previously [66], [67]. In brief, 10 µg of *in vitro*-synthesized HCV RNA was mixed with 5×10^6^ Huh7.5 cells in a 2-mm cuvette and pulsed twice at 1.4 kV and 25 µF. Cells were seeded into 12-well plates or 25-cm^2^ flasks, and passaged at 3-to 4-day intervals posttransfection by trypsinization and reseeding with a 1∶3 to 1∶4 split into fresh culture vessels. When infectivity reached ∼50%, as was monitored by immunofluorescent staining with anti HCV core protein mouse monoclonal antibody (Affinity BioReagents, USA) and Cy2-conjugated goat anti-mouse IgG (Jackson ImmunoResearch Laboratories, USA) (see “visualizing HCV infected cells (infectivity assay)” below), the mixed culture (of uninfected and HCV infected cells in 1∶1 ratio) was taken for cytotoxicity assays.

### Fluorescence/Immunofluorescence Microscopy

#### Confocal fluorescence microscopy analysis of T-REx HEK293 cells inducibly expressing EGFP-full NS3-4A and constitutively expressing mCherry-NS3 activated or uncleavable MazF

1×10^5^ Tet-NS3/activated MazF or Tet-NS3/uncleavable MazF cells were seeded on poly-L-lysine coated cover-slips in a 24 well-plate. 12 h later, the cells were supplemented with 1 µg/ml of tetracycline, or left untreated. 24 h later, the cells were fixed with 4% formaldehyde in PBS.

Uninduced cells were permeabilized with Triton X-100 (0.1% in PBS) for 5 minutes, blocked with 90% fetal calf serum/10% PBS at room temperature for 25 minutes, incubated with 1∶200 diluted rabbit-polyclonal anti-calnexin antibody (Sigma, USA) as primary antibody for 1 h and followed by 1∶200 diluted Cy2-conjugated anti-rabbit IgG (Jackson ImmunoResearch Laboratories, USA) secondary antibody for 30 minutes. Nuclei of induced and uninduced cells were then stained by Hoechst 33258 for 1 h at room temperature. Slides were washed with PBS, mounted in Mowiol 4-88 solution (Calbiochem, USA) (immunostained uninduced cells) or ImmuGlo Mounting Medium (IMMCO Diagnostics, USA) (induced Tet-NS3/uncleavable MazF cells) and examined using a Zeiss LSM 510 META laser scanning confocal microscope.

#### Visualizing T-REx HEK293 cells inducibly expressing EGFP-full NS3-4A (supplemented with different tetracycline concentrations) and constitutively expressing mCherry-NS3 activated MazF or mCherry-uncleavable MazF

1×10^5^ Tet-NS3/activated MazF or Tet-NS3/uncleavable MazF cells were seeded on poly-L-lysine coated cover-slips in a 24 well-plate. 12 h later, cells were supplemented with 10 ng/ml or 1000 ng/ml of tetracycline, or left untreated. 36 h later, cells were fixed with 4% formaldehyde in PBS. Following nuclear staining by Hoechst 33258 for 1 h at room temperature, Slides were washed with PBS, mounted in ImmuGlo Mounting Medium and examined using a fluorescence microscope.

#### Visualizing HEK293 cells infected with recombinant adenovirus encoding for mCherry-NS3 activated MazF or mCherry-uncleavable MazF

3×10^5^ HEK293 cells were seeded per well in 6 wells plate. When the culture reached 90% confluence, the growth medium was replaced by fresh medium containing 10 fold dilutions of recombinant adenoviruses encoding for mCherry-NS3 activated MazF or mCherry-uncleavable MazF, starting from 2.5×10^6^ PFU per well. After 36 h, cells were fixed with 4% formaldehyde in PBS and examined using a fluorescence microscope.

#### Visualizing HCV infected cells (infectivity assay)

Huh7.5 cells infected with HCV HJ3-5 chimeric virus were seeded into 8-well chamber slides (Nalge Nunc, USA). After 24 h, cells were fixed and permeabilized with 1∶1 acetone/methanol mixture and stained with 1∶300 diluted mouse monoclonal antibody C7-50 (Affinity BioReagents, USA) specific for the HCV core protein followed by staining with 1∶100 diluted Cy2-conjugated goat anti-mouse IgG (Jackson ImmunoResearch Laboratories, USA). Nuclei were then stained with DAPI (Sigma, Israel) and slides were washed with PBS, mounted (SouthernBiotech, USA) and examined using a fluorescence microscope.

#### Visualizing zymoxin-treated mixed culture of uninfected and HCV infected cells

See “Cytotoxicity assay of recombinant adenoviruses encoding for mCherry-NS3 activated MazF or mCherry-uncleavable MazF on a mixed culture of uninfected and HCV-infected Huh7.5 cells” below.

### Colony formation assay

7.5×10^5^ HEK293 T-REx cells were seeded per well in 6 wells plate. 24 h later, cells were transfected with 2 µg of the plasmids “pmCherry-NS3 activated MazF”, “pmCherry” or “pEGFP-MazF” encoding for mCherry-NS3 activated MazF, mCherry (only the fluorescent protein) or EGFP- MazF (where MazF is not fused to its inhibitory peptide), respectively. Transfection was carried out using FuGENE 6 reagent (Roche, Germany) according to the manufacturer instructions. After 48 h, transfection efficiency was assessed by fluorescence microscopy and was determined as equal between the plasmids. Transfected cells were then trypsinized, counted and seeded in 3 fold dilutions (starting from 150,000 cells/well) in 6 well plates and were incubated for 10 days in the presence of 1 mg/ml of G418 (to which all the three plasmids confer resistance). Surviving colonies were fixed with 4% formaldehyde in PBS and stained with Giemsa (Sigma, USA). Number of surviving colonies from wells that were seeded with 5556 cells was determined by manual counting.

### Cell-viability assay

The cell-killing activities of NS3-activated MazF and uncleavable MazF zymoxins were measured by a Thiazolyl Blue Tetrazoliam Bromide (MTT) assay as described below:

#### Cytotoxicity assay of intracellularly expressed mCherry-NS3 activated MazF or mCherry-uncleavable MazF in T-REx HEK293 cells inducibly expressing EGFP-Full NS3-4A

Tet-inducible full NS3-4A, Tet-NS3/activated MazF or Tet-NS3/uncleavable MazF cells were seeded in 96 well plates (2×10^4^ cells per well). After 24 h, cells were supplemented with 3 fold dilutions of tetracycline starting with concentration of 1000 ng/ml, or left untreated. 72 h later, the media was replaced by fresh media (100 µl per well) containing 1 mg/ml MTT (Thiazolyl Blue Tetrazoliam Bromide (Sigma, Israel) dissolved in PBS) reagent and the cells were incubated for further 30 minutes. MTT-formazan crystals were dissolved by the addition of extraction solution (20% SDS, 50% N, N-Dimethyl Formamide (DMF), pH 4.7) (100 µl per well) and incubation for 16 h at 37°C. Absorbance at 570 nm was recorded on an automated microtiter plate reader. The results were expressed as percentage of living cells relatively to the untreated controls.

#### Cytotoxicity assay of recombinant adenoviruses encoding for mCherry-NS3 activated MazF or mCherry-uncleavable MazF on full NS3-4A expressing Huh7.5 cells

1×10^4^ wild-type or EGFP-full NS3-4A expressing Huh7.5 cells [10] were seeded per well in 96 well plates. After 24 h, growth media were replaced by fresh media containing recombinant adenoviruses encoding for mCherry-NS3 activated MazF or mCherry-uncleavable MazF at indicated multiplicity of infection (MOI) ratios. Control cells remained untreated. Four days post infection, the media was replaced by fresh media (100 µl per well) containing 1 mg/ml MTT (except in representative wells in which cells were fixed and microscopically examined) and the cells were incubated for further 60 minutes. The next steps were identical to theses described above.

#### Cytotoxicity assay of recombinant adenoviruses encoding for mCherry-NS3 activated MazF or mCherry-uncleavable MazF on a mixed culture of uninfected and HCV-infected Huh7.5 cells

Uninfected Huh7.5 cells and mixed culture of HCV infected and uninfected cells at 1∶1 ratio (50% infected culture) were seeded in 96-well plates (1×10^4^ cells/well). After 24 h, cells were treated with recombinant adenoviruses (at MOI of ∼3) encoding for mCherry-NS3 activated MazF or mCherry-uncleavable MazF zymoxins. Control cells remained untreated. 72 h later, the media was replaced by fresh media (100 µl per well) containing 1 mg/ml MTT (except in representative wells in which cells were fixed and microscopically examined) and the cells were incubated for further 60 minutes. The next steps were identical to these described above.

For HCV-infection immunofluorescence analysis, 3×10^4^ cells from the HCV infected and uninfected mixed culture were seeded per well into 8-well chamber slides (Nalge Nunc, USA). 24 h later, cells were treated with recombinant adenoviruses (MOI of ∼3) encoding for the mCherry-NS3 activated MazF or mCherry-uncleavable MazF zymoxins. Control cells were remained untreated. 72 h post treatment, cells were fixed and permeabilized with 1∶1 acetone/methanol mixture and stained with 1∶300 diluted mouse monoclonal antibody C7-50 (Affinity BioReagents, USA) specific for the HCV core protein followed by staining with 1∶100 diluted FITC-conjugated goat anti-mouse IgG (Jackson ImmunoResearch Laboratories, USA). Nuclei were then stained with DAPI and slides were mounted (SouthernBiotech, USA) and examined using a fluorescence microscope. For each treatment, evaluation of the fraction of the HCV-infected cells from the general cell population was performed by dividing the number of the green, HCV-core positive cells by the general number of cells (DAPI stained) from five representative microscopic fields.

### [^3^H]-leucine incorporation assay

1×10^5^ Tet-NS3/activated MazF or Tet-NS3/uncleavable MazF cells were seeded per well in 24-wells plate. 24 or 48 h later, cells were supplemented with tetracycline to a final concentration of 1000 ng/ml, or left untreated. 72 h after seeding, cells were supplemented with [^3^H]-leucine (Perkin Elmer, USA) to a final concentration of 1 µCi/ml and returned to incubation. After 6 h, cells were scraped, washed with PBS and lysed by four freeze/thaw cycles. 7 µg total protein form the lysate of each treatment were then add to a solution containing PBS and a final concentration of 150 µg bovine serum albumin (BSA) in a total volume of 75 µl. The solution was then mixed with an identical volume of ice-cold 10% trichloro acetic acid (TCA). Mixtures were then incubated on ice for 30 minutes and centrifuged for 10 minutes at 20,000 g, 4°C, after which the pellet was washed with ice-cold 5% TCA, followed by washing with ice-cold 80% ethanol. The pellet was then dissolved in 300 µl of 0.1 M NaOH, transferred to a scintillation tube and neutralized with 200 µl 1 M HCl. 4 ml of scintillation liquid was added and radioactivity was counted by a beta-counter device.

## Supporting Information

Figure S1 **Fluorescence microscopy analysis of adenovirus producing foci.** 3×10^5^ HEK293 cells were seeded per well in 6 wells plate. When reached 90% confluence, cells were infected with 10 fold dilutions of recombinant adenoviruses encoding for mCherry-NS3 activated MazF or mCherry-uncleavable MazF, starting from 2.5×10^6^ PFU per well. After 36 h, cells were fixed and examined under a fluorescence microscope. Red fluorescent adenovirus-producing foci from wells infected with 2.5×10^3^ PFU are shown. The bar represents 200 µm.
(DOC)

Table S1 **Oligonucleotides that have been used in this study.**
(DOC)

Text S1 **Amino-acid sequences of MazF based zymoxins.**
(DOC)

Text S2 **Construction and propagation of recombinant adenoviral vectors.**
(DOC)

## References

[1] Pawlotsky JM (2011). Treatment failure and resistance with direct-acting antiviral drugs against hepatitis C virus.. Hepatology.

[2] Moradpour D, Penin F, Rice CM (2007). Replication of hepatitis C virus.. Nat Rev Microbiol.

[3] Suzuki T, Ishii K, Aizaki H, Wakita T (2007). Hepatitis C viral life cycle.. Adv Drug Deliv Rev.

[4] De Francesco R, Carfi A (2007). Advances in the development of new therapeutic agents targeting the NS3-4A serine protease or the NS5B RNA-dependent RNA polymerase of the hepatitis C virus.. Adv Drug Deliv Rev.

[5] Bartenschlager R (1999). The NS3/4A proteinase of the hepatitis C virus: unravelling structure and function of an unusual enzyme and a prime target for antiviral therapy.. J Viral Hepat.

[6] Neurath H, Walsh KA (1976). Role of proteolytic enzymes in biological regulation (a review).. Proc Natl Acad Sci U S A.

[7] Richter C, Tanaka T, Yada RY (1998). Mechanism of activation of the gastric aspartic proteinases: pepsinogen, progastricsin and prochymosin.. Biochem J.

[8] Donepudi M, Grutter MG (2002). Structure and zymogen activation of caspases.. Biophys Chem.

[9] Davie EW (2003). A brief historical review of the waterfall/cascade of blood coagulation.. J Biol Chem.

[10] Shapira A, Gal-Tanamy M, Nahary L, Litvak-Greenfeld D, Zemel R (2011). Engineered toxins “zymoxins” are activated by the HCV NS3 protease by removal of an inhibitory protein domain.. PLoS One.

[11] Gerdes K, Moller-Jensen J, Ebersbach G, Kruse T, Nordstrom K (2004). Bacterial mitotic machineries.. Cell.

[12] Couturier M, Bahassi el M, Van Melderen L (1998). Bacterial death by DNA gyrase poisoning.. Trends Microbiol.

[13] Engelberg-Kulka H, Glaser G (1999). Addiction modules and programmed cell death and antideath in bacterial cultures.. Annu Rev Microbiol.

[14] Gerdes K, Christensen SK, Lobner-Olesen A (2005). Prokaryotic toxin-antitoxin stress response loci.. Nat Rev Microbiol.

[15] Hayes F (2003). Toxins-antitoxins: plasmid maintenance, programmed cell death, and cell cycle arrest.. Science.

[16] Yarmolinsky MB (1995). Programmed cell death in bacterial populations.. Science.

[17] Magnuson RD (2007). Hypothetical functions of toxin-antitoxin systems.. J Bacteriol.

[18] Mittenhuber G (1999). Occurrence of mazEF-like antitoxin/toxin systems in bacteria.. J Mol Microbiol Biotechnol.

[19] Pandey DP, Gerdes K (2005). Toxin-antitoxin loci are highly abundant in free-living but lost from host-associated prokaryotes.. Nucleic Acids Res.

[20] Van Melderen L, Saavedra De Bast M (2009). Bacterial toxin-antitoxin systems: more than selfish entities?. PLoS Genet.

[21] Engelberg-Kulka H, Hazan R, Amitai S (2005). mazEF: a chromosomal toxin-antitoxin module that triggers programmed cell death in bacteria.. J Cell Sci.

[22] Yamaguchi Y, Inouye M (2009). mRNA interferases, sequence-specific endoribonucleases from the toxin-antitoxin systems.. Prog Mol Biol Transl Sci.

[23] Engelberg-Kulka H, Amitai S, Kolodkin-Gal I, Hazan R (2006). Bacterial programmed cell death and multicellular behavior in bacteria.. PLoS Genet.

[24] Kamada K, Hanaoka F, Burley SK (2003). Crystal structure of the MazE/MazF complex: molecular bases of antidote-toxin recognition.. Mol Cell.

[25] Li GY, Zhang Y, Chan MC, Mal TK, Hoeflich KP (2006). Characterization of dual substrate binding sites in the homodimeric structure of Escherichia coli mRNA interferase MazF.. J Mol Biol.

[26] Inouye M (2006). The discovery of mRNA interferases: implication in bacterial physiology and application to biotechnology.. J Cell Physiol.

[27] Shimazu T, Degenhardt K, Nur EKA, Zhang J, Yoshida T (2007). NBK/BIK antagonizes MCL-1 and BCL-XL and activates BAK-mediated apoptosis in response to protein synthesis inhibition.. Genes Dev.

[28] Anderie I, Schulz I, Schmid A (2007). Characterization of the C-terminal ER membrane anchor of PTP1B.. Exp Cell Res.

[29] Shaner NC, Campbell RE, Steinbach PA, Giepmans BN, Palmer AE (2004). Improved monomeric red, orange and yellow fluorescent proteins derived from Discosoma sp. red fluorescent protein.. Nat Biotechnol.

[30] Wolk B, Sansonno D, Krausslich HG, Dammacco F, Rice CM (2000). Subcellular localization, stability, and trans-cleavage competence of the hepatitis C virus NS3-NS4A complex expressed in tetracycline- regulated cell lines.. J Virol.

[31] Moradpour D, Gosert R, Egger D, Penin F, Blum HE (2003). Membrane association of hepatitis C virus nonstructural proteins and identification of the membrane alteration that harbors the viral replication complex.. Antiviral Res.

[32] Brass V, Berke JM, Montserret R, Blum HE, Penin F (2008). Structural determinants for membrane association and dynamic organization of the hepatitis C virus NS3-4A complex.. Proc Natl Acad Sci U S A.

[33] Egger D, Wolk B, Gosert R, Bianchi L, Blum HE (2002). Expression of hepatitis C virus proteins induces distinct membrane alterations including a candidate viral replication complex.. J Virol.

[34] Wolk B, Buchele B, Moradpour D, Rice CM (2008). A dynamic view of hepatitis C virus replication complexes.. J Virol.

[35] He TC, Zhou S, da Costa LT, Yu J, Kinzler KW (1998). A simplified system for generating recombinant adenoviruses.. Proc Natl Acad Sci U S A.

[36] Zheng C, Goldsmith CM, O'Connell BC, Baum BJ (2000). Adenoviral vector cytotoxicity depends in part on the transgene encoded.. Biochem Biophys Res Commun.

[37] Wersto RP, Rosenthal ER, Seth PK, Eissa NT, Donahue RE (1998). Recombinant, replication-defective adenovirus gene transfer vectors induce cell cycle dysregulation and inappropriate expression of cyclin proteins.. J Virol.

[38] Teramoto S, Johnson LG, Huang W, Leigh MW, Boucher RC (1995). Effect of adenoviral vector infection on cell proliferation in cultured primary human airway epithelial cells.. Hum Gene Ther.

[39] Teramoto S, Matsuse T, Matsui H, Ohga E, Ishii T (1999). Recombinant E1-deleted adenovirus vector induces apoptosis in two lung cancer cell lines.. Eur Respir J.

[40] Yi M, Ma Y, Yates J, Lemon SM (2007). Compensatory mutations in E1, p7, NS2, and NS3 enhance yields of cell culture-infectious intergenotypic chimeric hepatitis C virus.. J Virol.

[41] Bartenschlager R, Sparacio S (2007). Hepatitis C virus molecular clones and their replication capacity in vivo and in cell culture.. Virus Res.

[42] Tellinghuisen TL, Evans MJ, von Hahn T, You S, Rice CM (2007). Studying hepatitis C virus: making the best of a bad virus.. J Virol.

[43] Thompson AJ, McHutchison JG (2009). Review article: investigational agents for chronic hepatitis C.. Aliment Pharmacol Ther.

[44] Greene WC, Debyser Z, Ikeda Y, Freed EO, Stephens E (2008). Novel targets for HIV therapy.. Antiviral Res.

[45] Wainberg MA (2009). Perspectives on antiviral drug development.. Antiviral Res.

[46] Hsu JT, Wang HC, Chen GW, Shih SR (2006). Antiviral drug discovery targeting to viral proteases.. Curr Pharm Des.

[47] Patick AK, Potts KE (1998). Protease inhibitors as antiviral agents.. Clin Microbiol Rev.

[48] Gal-Tanamy M, Zemel R, Bachmatov L, Jangra RK, Shapira A (2010). Inhibition of protease-inhibitor-resistant hepatitis C virus replicons and infectious virus by intracellular intrabodies.. Antiviral Res.

[49] Trahtenherts A, Gal-Tanamy M, Zemel R, Bachmatov L, Loewenstein S (2008). Inhibition of hepatitis C virus RNA replicons by peptide aptamers.. Antiviral Res.

[50] Gal-Tanamy M, Zemel R, Berdichevsky Y, Bachmatov L, Tur-Kaspa R (2005). HCV NS3 serine protease-neutralizing single-chain antibodies isolated by a novel genetic screen.. J Mol Biol.

[51] Varshavsky A (1995). The N-end rule.. Cold Spring Harb Symp Quant Biol.

[52] Falnes PO, Welker R, Krausslich HG, Olsnes S (1999). Toxins that are activated by HIV type-1 protease through removal of a signal for degradation by the N-end-rule pathway.. Biochem J.

[53] Johnson RJ, Lin SR, Raines RT (2006). A ribonuclease zymogen activated by the NS3 protease of the hepatitis C virus.. Febs J.

[54] Plainkum P, Fuchs SM, Wiyakrutta S, Raines RT (2003). Creation of a zymogen.. Nat Struct Biol.

[55] Turcotte RF, Raines RT (2008). Design and Characterization of an HIV-Specific Ribonuclease Zymogen.. AIDS Res Hum Retroviruses.

[56] Jucovic M, Walters FS, Warren GW, Palekar NV, Chen JS (2008). From enzyme to zymogen: engineering Vip2, an ADP-ribosyltransferase from Bacillus cereus, for conditional toxicity.. Protein Eng Des Sel.

[57] Law SK, Wang RR, Mak AN, Wong KB, Zheng YT (2010). A switch-on mechanism to activate maize ribosome-inactivating protein for targeting HIV-infected cells.. Nucleic Acids Res.

[58] Hsu EC, Hsi B, Hirota-Tsuchihara M, Ruland J, Iorio C (2003). Modified apoptotic molecule (BID) reduces hepatitis C virus infection in mice with chimeric human livers.. Nat Biotechnol.

[59] Willis A, Jung EJ, Wakefield T, Chen X (2004). Mutant p53 exerts a dominant negative effect by preventing wild-type p53 from binding to the promoter of its target genes.. Oncogene.

[60] Rebe C, Cathelin S, Launay S, Filomenko R, Prevotat L (2007). Caspase-8 prevents sustained activation of NF-kappaB in monocytes undergoing macrophagic differentiation.. Blood.

[61] Li P, Nijhawan D, Budihardjo I, Srinivasula SM, Ahmad M (1997). Cytochrome c and dATP-dependent formation of Apaf-1/caspase-9 complex initiates an apoptotic protease cascade.. Cell.

[62] Ham J, Babij C, Whitfield J, Pfarr CM, Lallemand D (1995). A c-Jun dominant negative mutant protects sympathetic neurons against programmed cell death.. Neuron.

[63] Yan N, Huh JR, Schirf V, Demeler B, Hay BA (2006). Structure and activation mechanism of the Drosophila initiator caspase Dronc.. J Biol Chem.

[64] Luo J, Deng ZL, Luo X, Tang N, Song WX (2007). A protocol for rapid generation of recombinant adenoviruses using the AdEasy system.. Nat Protoc.

[65] Blight KJ, McKeating JA, Rice CM (2002). Highly permissive cell lines for subgenomic and genomic hepatitis C virus RNA replication.. J Virol.

[66] Yi M, Lemon SM (2004). Adaptive mutations producing efficient replication of genotype 1a hepatitis C virus RNA in normal Huh7 cells.. J Virol.

[67] Yi M, Villanueva RA, Thomas DL, Wakita T, Lemon SM (2006). Production of infectious genotype 1a hepatitis C virus (Hutchinson strain) in cultured human hepatoma cells.. ProcNatlAcadSciUSA.

