# Supplementary material for: Removal of Hepatitis C Virus-Infected Cells by a Zymogenized Bacterial Toxin
Source: PLoS One. 2012 Feb 16;7(2):e32320. doi: 10.1371/journal.pone.0032320 (PMC3281143; doi:10.1371/journal.pone.0032320)
Supplement: Figure S1 — Fluorescence microscopy analysis of adenovirus producing foci. 3×10^5^ HEK293 cells were seeded per well in 6 wells plate. When reached 90% confluence, cells were infected with 10 fold dilutions of recombinant adenoviruses encoding for mCherry-NS3 activated MazF or mCherry-uncleavable MazF, starting from 2.5×10^6^ PFU per well. After 36 h, cells were fixed and examined under a fluorescence microscope. Red fluorescent adenovirus-producing foci from wells infected with 2.5×10^3^ PFU are shown. The bar represents 200 µm. (DOC) [file pone.0032320.s001.doc]

**Supporting Figure S1. Fluorescence microscopy analysis of adenovirus producing foci.** 3 × 10^5^ HEK 293 cells were seeded per well in 6 wells plate. When reached 90% confluence, cells were infected with 10 fold dilutions of recombinant adenoviruses encoding for mCherry-NS3 activated MazF or mCherry-uncleavable-MazF, starting from 2.5 × 10^6^ PFU per well. After 36 h, cells were fixed and examined under a fluorescence microscope. Red fluorescent adenovirus-producing foci from wells infected with 2.5 × 10^3^ PFU are shown. The bar represents 200µm.
