# Supplementary material for: Removal of Hepatitis C Virus-Infected Cells by a Zymogenized Bacterial Toxin
Source: PLoS One. 2012 Feb 16;7(2):e32320. doi: 10.1371/journal.pone.0032320 (PMC3281143; doi:10.1371/journal.pone.0032320)
Supplement: Table S1 — Oligonucleotides that have been used in this study. (DOC) [file pone.0032320.s002.doc]

| **Primer name** | **Sequence (5’ – 3’)** | **Orientation** | **Restriction sites underlined** | **Construct name** |
| --- | --- | --- | --- | --- |
| **40- clvmazf** | GGACTCAGATCTCGAGCATGGTAAGCCGATACGTACCCG | forward | *Xho*I | mCherry-NS3 activated MazF**;**  uncleavable- MazF |
| **41- clvmazf** | GAGCAACATACGGTCGTGTCATCTTCTTCAGAGCTTCCGCCAATCAGTACGTTAATTTTGGCTTTAATG | reverse |  | mCherry-NS3 activated MazF |
| **42- clvmazf** | CTTTTCGCGACCCGCCACCGCCCAGCGCACCAGTCCAGGAGTAGCTCATAGAGCAACATACGGTCGTGT | reverse |  | mCherry-NS3 activated MazF |
| **43-clvmazf** | GTTTTCCGGCGTGATGTCGTTGACTAGTTCAGCAAGCGTAAATACGGGCTCTTTTCGCGACCCGCCACCG | reverse |  | mCherry-NS3 activated MazF |
| **44-clvmazf** | CCAGACTTCCTTATCTTTCGGCTCTCCCCAGTCGATATTCTCGTGGAGGTTTTCCGGCGTGATGTCGTTG | reverse |  | mCherry-NS3 activated MazF |
| **45-clvmazf** | CTCAGTCCGGAACCTCCGCTACCGGATCCGCCGCCAGAACCGCCTGAGCTCCAGACTTCCTTATCTTTCG | reverse |  | mCherry-NS3 activated MazF |
| **46-clvmazf** | GCTCCGGCTGTCAGCACAGTTGCGACACACATATTCACAAGAAAGCTTCTCAGTCCGGAACCTCCGCTAC | reverse |  | mCherry-NS3 activated MazF |
| **47-clvmazf** | GTCGACTGCAGAATTCCTATGTGTTGCTATTGAACAGGAATCTGTAGCAAAGATATGCTCCGGCTGTCAGCACAGTTG | reverse | *Eco*RI | mCherry-NS3 activated MazF |
| **48- clvmazf** | CGTCAGATCCGCTAGCGCTACCGGTCGCCACCATGGTGAGCAAGGGCGAGGAGGATAAC | forward | *Nhe*I | mCherry-NS3 activated MazF;  mCherry |
| **49- clvmazf** | GCTTACCATGCTCGAGATCTGAGTCCGGCCGGACTTGTACAGCTCGTCCATGCCG | reverse | *Xho*I*, Bgl*II | mCherry-NS3 activated MazF;  mCherry |
| **50-unclmazf** | GCACTCATAGATCCGCCAGCTACGTCTTCTGTGTCAGCACCGCTTCCGCCAATCAGTACGTTAATTTTGG | reverse |  | mCherry-uncleavable- MazF |
| **51-unclmazf** | CGGGCTCTTTTCGCGACCCGCCACCGCCGGTCCAAGTGGCACTCATAGATCCGCCAGC | reverse | *Nru*I | mCherry-uncleavable- MazF |

**Supporting Table S1. Oligonucleotides that have been used in this study**
