# Supplementary material for: Removal of Hepatitis C Virus-Infected Cells by a Zymogenized Bacterial Toxin
Source: PLoS One. 2012 Feb 16;7(2):e32320. doi: 10.1371/journal.pone.0032320 (PMC3281143; doi:10.1371/journal.pone.0032320)
Supplement: Text S1 — Amino-acid sequences of MazF based zymoxins. (DOC) [file pone.0032320.s003.doc]

**Supporting Text S1. Amino-acid sequences of MazF based zymoxins.**

Indicated numbers refers to the amino acid sequence to their right. Legends are positioned under each sequence

mCherry-NS3 activated MazF

(1)MVSKGEEDNMAIIKEFMRFKVHMEGSVNGHEFEIEGEGEGRPYEGTQTAKLKVTKGGPLPFAWDILSPQFMYGSKAYVKHPADIPDYLKLSFPEGFKWERVMNFEDGGVVTVTQDSSLQDGEFIYKVKLRGTNFPSDGPVMQKKTMGWEASSERMYPEDGALKGEIKQRLKLKDGGHYDAEVKTTYKAKKPVQLPGAYNVNIKLDITSHNEDYTIVEQYERAEGRHSTGGMDELYK(2)SGRTQISS(3)MVSRYVPDMGDLIWVDFDPTKGSEQAGHRPAVVLSPFMYNNKTGMCLCVPCTTQSKGYPFEVVLSGQERDGVALADQVKSIAWRARGATKKGTVAPEELQLIKAKINVLI(4)GGS(5)SEEDDTTVCCSMSYSWTGAL(6)GGGGS(7)RKEPVFTLAELVNDITPENLHENIDWGEPKDKEVW(8)SSGGSGGGSGSGG(9)SGLRSFLVNMCVATVLTAGAYLCYRFLFNSNT

(1) mCherry; (2) linker; (3) MazF; (4) linker; (5) P10-P10’ NS3 cleavable NS5A/B junction sequence derived from HCV 2a genotype (strain JFH1); (6) linker; (7) Inhibitory peptide corresponding to MazE C-terminal 35 amino-acids; (8) linker; (9) The C-terminal ER membrane anchor of the tyrosine phosphatase PTP1B

mCherry-uncleavable MazF

(1)MVSKGEEDNMAIIKEFMRFKVHMEGSVNGHEFEIEGEGEGRPYEGTQTAKLKVTKGGPLPFAWDILSPQFMYGSKAYVKHPADIPDYLKLSFPEGFKWERVMNFEDGGVVTVTQDSSLQDGEFIYKVKLRGTNFPSDGPVMQKKTMGWEASSERMYPEDGALKGEIKQRLKLKDGGHYDAEVKTTYKAKKPVQLPGAYNVNIKLDITSHNEDYTIVEQYERAEGRHSTGGMDELYK(2)SGRTQISS(3)MVSRYVPDMGDLIWVDFDPTKGSEQAGHRPAVVLSPFMYNNKTGMCLCVPCTTQSKGYPFEVVLSGQERDGVALADQVKSIAWRARGATKKGTVAPEELQLIKAKINVLI(4)GGS(5)GADTEDVAGGSMSATWT(6)GGGGS(7)RKEPVFTLAELVNDITPENLHENIDWGEPKDKEVW(8)SSGGSGGGSGSGG(9)SGLRSFLVNMCVATVLTAGAYLCYRFLFNSNT

(1) mCherry; (2) linker; (3) MazF; (4) linker; (5) Mutated uncleavable NS5A/B junction sequence derived from HCV 1a genotype; (6) linker; (7) Inhibitory peptide corresponding to MazE C-terminal 35 amino-acids; (8) linker; (9) The C-terminal ER membrane anchor of the tyrosine phosphatase PTP1B
