# Supplementary material for: Removal of Hepatitis C Virus-Infected Cells by a Zymogenized Bacterial Toxin
Source: PLoS One. 2012 Feb 16;7(2):e32320. doi: 10.1371/journal.pone.0032320 (PMC3281143; doi:10.1371/journal.pone.0032320)
Supplement: Text S2 — Construction and propagation of recombinant adenoviral vectors. (DOC) [file pone.0032320.s004.doc]

**Supporting Text S2. Construction and propagation of recombinant adenoviral vectors**

Construction and propagation of recombinant human type 5 adenoviral vectors for gene delivery of the mCherry-NS3 activated MazF and mCherry-uncleavable MazF expression cassettes were carried out using the AdEasy system essentially as described in . Briefly, The DNA sequence of the mCherry-NS3 activated MazF and mCherry-uncleavable MazF expression cassettes was cloned into “pShuttle” vectors by standard recombinant DNA methods. The resulting vectors were digested with *Pme*I, purified, and 400ng of the digested vectors where mixed with 100ng of the plasmid pAdEasy-1. DNA mixture was electrophorated (in 2.0 mm cuvettes at 2,500V, 25 µF) into *E. coli* strain BJ5183 competent cells (Stratagene, USA). After phenotypic expression of 1 h at 37ºC, bacteria were seeded on LB agar plates containing 50 µg/ml of kanamycin and were grown at 37ºC over-night. 15 smallest colonies were picked, grown for 12 h in LB containing 25 µg/ml of kanamycin and plasmids form 5ml of each culture were purified by standard miniprep procedure. *Pac*I digested plasmids were analyzed by 0.8% TAE agarose gel electrophoresis. “Candidate” plasmids that yield a large fragment (near 30 kb), plus a smaller fragment of 3.0kb or 4.5kb, were transfomed into DH5α competent cells. Plasmids were isolated by standard miniprep procedure and were sequenced to confirm their predicted composition. For production of virus particles, the plasmids, which were denoted “pAdEasy-mCherry-NS3 activated MazF” and “pAdEasy- mCherry-uncleavable MazF” were isolated from chosen “positive” clones and were digested with *Pac*I. Digested DNA was purified using Zymoclean^TM^ Gel DNA Recovery Kit (ZYMO RESEARCH, USA) according to the manufacturer instructions. 1.5 µg of the purified, digested plasmids were used to transfect 50-70% confluence HEK293 cells in 60mm culture dish using the calcium-phosphate method. 7 to 10 days post-transfection, when cytopathic effect (CPE) was clearly observed, cells were collected by scraping them off the dish and pelleting them along with any floating cells in the culture. Pellet was washed once with PBS, suspended in 0.5ml PBS and was subjected to 4 cycles of freeze/thaw. Cell debris were precipitated by brief centrifugation and 300µl of the supernatant that contains virus particles were used to infect 70% confluent HEK293 cells in two 60mm dishes (first amplification “cycle”). When a third to half of the cells is detached (usually after 3-5 days), virus particle were released by freeze/thaw cycles as described, and a second amplification cycle was performed, as described, by infecting 70% confluent HEK293 cells in two 100mm dishes. Supernatant containing viruses was kept in -80ºC. Viral titers were determined by end-point dilution assay: HEK293 cells were grown to about 70% confluence in 96-wells plates. The recombinant adenovirus stock solution was 10 fold serially diluted to a concentrations range of 10^-3^-10^-10^ into growth medium. 10×100 μL of each dilution were added to 10 wells in the 96-well plate. After cultured for 10 days, plaque forming units (PFU) in one ml of the concentrated sample was calculated according to the formula of titer (pfu/mL) = 10^(x + 0.8)^, when X represents the sum of the fractions of CPE positive wells for each dilution (10 of 10 wells with CPE is calculated as ”1”) .

**Supplementary references**

1. He TC, Zhou S, da Costa LT, Yu J, Kinzler KW, et al. (1998) A simplified system for generating recombinant adenoviruses. Proc Natl Acad Sci U S A 95: 2509-2514.

2. Luo J, Deng ZL, Luo X, Tang N, Song WX, et al. (2007) A protocol for rapid generation of recombinant adenoviruses using the AdEasy system. Nat Protoc 2: 1236-1247.
